# Human Inborn Errors of Immunity: 2019 Update on the Classification from the International Union of Immunological Societies Expert Committee

**DOI:** 10.1007/s10875-019-00737-x

**Published:** 2020-01-17

**Authors:** Stuart G. Tangye, Waleed Al-Herz, Aziz Bousfiha, Talal Chatila, Charlotte Cunningham-Rundles, Amos Etzioni, Jose Luis Franco, Steven M. Holland, Christoph Klein, Tomohiro Morio, Hans D. Ochs, Eric Oksenhendler, Capucine Picard, Jennifer Puck, Troy R. Torgerson, Jean-Laurent Casanova, Kathleen E. Sullivan

**Affiliations:** 1 Garvan Institute of Medical Research, Darlinghurst, Sydney, NSW 2010 Australia; 2 Faculty of Medicine, St Vincent’s Clinical School, UNSW, Sydney, NSW Australia; 3 Department of Pediatrics, Faculty of Medicine, Kuwait University, Kuwait City, Kuwait; 4 King Hassan II University, Laboratoire d’Immunologie Clinique, d’Inflammation et d’Allergy LICIA at Faculty of Medicine and Pharmacy, Clinical Immunology Unit, Pediatric Infectiouse Disease Department, Children’s Hospital, Ibn Rochd University Hospital, Casablanca, Morocco; 5 Division of Immunology, Children’s Hospital Boston, Boston, MA USA; 6 Departments of Medicine and Pediatrics, Mount Sinai School of Medicine, New York, NY USA; 7 Ruth’s Children’s Hospital-Technion, Haifa, Israel; 8 Grupo de Inmunodeficiencias Primarias, Facultad de Medicina, Universidad de Antioquia UdeA, Medellin, Colombia; 9 Laboratory of Clinical Immunology & Microbiology, National Institute of Allergy and Infectious Diseases, National Institutes of Health, Bethesda, MD USA; 10 Dr von Hauner Children’s Hospital, Ludwig-Maximilians-University Munich, Munich, Germany; 11 Department of Pediatrics and Developmental Biology, Tokyo Medical and Dental University (TMDU), Tokyo, Japan; 12 Department of Pediatrics, University of Washington and Seattle Children’s Research Institute, Seattle, WA USA; 13 Department of Clinical Immunology, Hôpital Saint-Louis, APHP, University Paris Diderot, Sorbonne Paris Cité, Paris, France; 14 Study Center for Primary Immunodeficiencies, Necker Hospital for Sick Children, APHP, Paris, France; 15 Paris University, Laboratory of Lymphocyte Activation and Susceptibility to EBV, INSERM UMR1163, Imagine Institute, Necker Hospital for Sick Children, Paris, France; 16 Department of Pediatrics, University of California San Francisco and UCSF Benioff Children’s Hospital, San Francisco, CA USA; 17 St. Giles Laboratory of Human Genetics of Infectious Diseases, Rockefeller Branch, The Rockefeller University, New York, NY USA; 18 Howard Hughes Medical Institute, New York, NY USA; 19 Laboratory of Human Genetics of Infectious Diseases, Necker Branch, INSERM UMR1163, Imagine Institute, Necker Hospital for Sick Children, Paris University, Paris, France; 20 Pediatric Hematology-Immunology Unit, Necker Hospital for Sick Children, Assistance Publique-Hôpitaux de Paris (APHP), Paris, France; 21 Division of Allergy Immunology, Department of Pediatrics, The Children’s Hospital of Philadelphia, University of Pennsylvania Perelman School of Medicine, Philadelphia, PA USA

**Keywords:** IUIS, primary immune deficiency, inborn errors of immunity, immune dysregulation, autoinflammatory disorders, next-generation sequencing

## Abstract

We report the updated classification of Inborn Errors of Immunity/Primary Immunodeficiencies, compiled by the International Union of Immunological Societies Expert Committee. This report documents the key clinical and laboratory features of 430 inborn errors of immunity, including 64 gene defects that have either been discovered in the past 2 years since the previous update (published January 2018) or were characterized earlier but have since been confirmed or expanded upon in subsequent studies. The application of next-generation sequencing continues to expedite the rapid identification of novel gene defects, rare or common; broaden the immunological and clinical phenotypes of conditions arising from known gene defects and even known variants; and implement gene-specific therapies. These advances are contributing to greater understanding of the molecular, cellular, and immunological mechanisms of disease, thereby enhancing immunological knowledge while improving the management of patients and their families. This report serves as a valuable resource for the molecular diagnosis of individuals with heritable immunological disorders and also for the scientific dissection of cellular and molecular mechanisms underlying inborn errors of immunity and related human diseases.

Inborn errors of immunity, also referred to as primary immunodeficiencies, manifest as increased susceptibility to infectious diseases, autoimmunity, autoinflammatory diseases, allergy, and/or malignancy. These conditions are caused by monogenic germline mutations that result in loss of expression, loss-of-function (LOF; amorphic/hypomorphic), or gain-of-function (GOF; hypermorphic) of the encoded protein [1, 2]. Heterozygous lesions may underlie autosomal dominant traits by GOF, haploinsufficiency, or negative dominance. Biallelic lesions typically cause autosomal recessive traits by LOF of the encoded protein (rarely GOF), while X-linked recessive traits arise from LOF of genes on the X chromosome, either in the hemizygous state in males or in the homozygous state in females. Rare X-linked dominant traits can also arise from LOF or GOF variants. This results in aberrant immunity due to the critical roles of these proteins in the development, maintenance and function of cells of the immune system, or cells other than leukocytes that contribute to immunity, during homeostasis and in response to external (e.g., infectious agents or environmental antigens) and internal (e.g., cytokines, self-antigens and cancer cells) stimuli [3–5]. Inborn errors of immunity were traditionally considered to be rare diseases, affecting ~ 1 in 10,000 to 1 in 50,000 births. However, with ongoing discovery of novel inborn errors of immunity (Fig. 1a) and improved definition of clinical phenotypes [6–8], the collective prevalence of these conditions is more likely to be at least 1/1000–1/5000 [9]. Indeed, more common inborn errors have recently been described [10]. Regardless of their exact incidence and prevalence, inborn errors of immunity represent an unprecedented model to link defined monogenic defects with clinical phenotypes of immune dysregulation, in a broad sense of the term. As a committee, we are aware that human immunity involves cells other than circulating or tissue leukocytes and that it can be scaled up from the immune system to the whole organism. Inborn errors of immunity have unequivocally revealed non-redundant roles of single genes and their products in immune function [3, 4, 6–8], formed the basis of improved mechanism-based therapies for the immunopathology underlying many diseases [8, 11], established immunological paradigms representing the foundations of basic, clinical and translational immunology [3–5, 9, 12–14], and provided insights into the molecular pathogenesis of more common diseases [9, 15]. Clear examples of these include:

- The initial description by Bruton of X-linked agammaglobulinemia (XLA) and the ability to treat this condition with antibody replacement therapy (the mainstay treatment for antibody deficiency diseases such as CVID) [16]
- The discovery of mutations in *BTK* [12] and the subsequent development of BTK-inhibitors such as ibrutinib for the treatment of B cell malignancies [14]
- Progressive CD4 T cell deficiency explains opportunistic infections secondary to HIV infection [9].

**Fig. 1:**
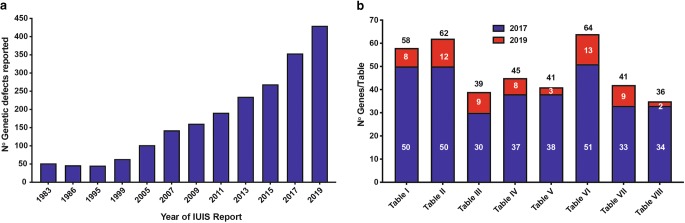
Rate of discovery of novel inborn errors of immunity: 1983–2019. **a** The number of genetic defects underlying monogenic immune disorders as reported by the IUIS/WHO committee in the indicated year. **b** The number of pathogenic gene variants listed in each table by the IUIS committee. Report published in 2017, and the number of new genes for each table contained in this report (red bars). The numbers in each column correspond to the number of genes reported in the 2017 IUIS update (blue bars) [1, 2], the number of new genes for each table contained in this report (red bars), and the total number of genes for each table. Note: only data for Tables 1, 2, 3, 4, 5, 6, 7, and 8 are shown, because Table 9 (bone marrow failure) is a new addition to the current report.

Thus, the study of inborn errors of immunity has provided profound advances in the practice of precision molecular medicine.

Since the early 1950s, when XLA was one of the first primary immune deficiencies to be described [16], clinical immunology has leveraged advances in the development of new methods to expedite the identification of defects of the immune system and the cellular, molecular, and genetic aberrations underlying these conditions. Indeed, the completion of the Human Genome Project in the early 2000s, coupled with rapid developments in next generation DNA sequencing (NGS) technologies, enabled the application of cost-effective and time-efficient sequencing of targeted gene panels, whole exomes, or whole genomes to cohorts of patients suspected of having a monogenic explanation for their disease. These platforms have led to a quantum leap in the identification and diagnosis of previously undefined genetically determined defects of the immune system (Fig. 1a, b; [6–8]).

The International Union of Immunological Societies Expert Committee of Inborn Errors of Immunity comprises pediatric and adult clinical immunologists, clinician/scientists and researchers in basic immunology from across the globe (https://iuis.org/committees/iei/). A major objective and responsibility of the committee is to provide the clinical and research communities with an update of genetic causes of immune deficiency and dysregulation. The committee has existed since 1970 and has published an updated report approximately every 2 years to inform the field of these advances (Fig. 1a). In March 2019, the committee met in New York to discuss and debate the inclusion of genetic variants published over the preceding 2 years (since June 2017) [1, 2], as well as gene mutations that had appeared in the literature earlier but, based on newly available evidence, were now substantiated (Fig. 1b).

Rather than simply including every gene variant reported, the committee applies very stringent criteria such that only those genes with convincing evidence of disease pathogenicity are classified as causes of novel inborn errors of immunity [17]. The Committee makes informed judgments for including new genetic causes of immunological conditions based on what we believe is most useful for practitioners caring for patients. Our current, and continuously evolving, practice is that criteria for inclusion can be met by several ways, for instance peer-reviewed publication of (1) multiple cases from unrelated kindreds, including detailed immunologic data, or (2) very few cases, or even a single case (see below), for whom compelling mechanistic/pathogenic data is also provided, generally from parallel studies in an animal or cell culture model.

Herein, we provide this latest update. The inborn errors of immunity are listed in 10 tables: Combined immunodeficiencies (Table 1), Combined immunodeficiencies with syndromic features (Table 2), Predominantly antibody deficiencies (Table 3), Diseases of immune dysregulation (Table 4), Congenital defects of phagocytes (Table 5), Defects in intrinsic and innate immunity (Table 6), Autoinflammatory diseases (Table 7), Complement deficiencies (Table 8), and Phenocopies of inborn errors of immunity (Table 10) (Fig. 1b). Since the last update (published January 2018) [1, 2], we have added a new table to consolidate genes that cause bone marrow failure (Table 9). Our division into phenotypes does not imply that the presentation is homogeneous. Rather, we recognize that substantial phenotypic and clinical heterogeneity exists within groups of patients with mutations in the same gene and even between individuals from the same pedigree with the identical gene mutation. To simplify the classification, each disorder has been listed only once, although distinct disorders due to mutations in the same gene, but with different modes of inheritance and pathogenic mechanisms are listed individually. Thus, several genes appear more than once in this update (some examples are listed below). Sub-divisions within each table segregate groups of disorders into coherent phenotypic sets. OMIM numbers are also provided within each table. If a OMIM number has not yet been issued for a particular genetic condition, then the number provided generally refers to the OMIM for that gene. Beneath each table, the new disorders added to this update are highlighted for easy reference.

**Table 1:** Immunodeficiencies affecting cellular and humoral immunity

Disease	Genetic defect	Inheritance	OMIM	T cells	B cells	Ig	Associated features
1. T-B+ severe combined immune deficiency (SCID)							
γc deficiency (common gamma chain SCID, CD132 deficiency)	*IL2RG*	XL	308380	Very low	Normal to high	Low	Low NK
JAK3 deficiency	*JAK3*	AR	600173	Very low	Normal to high	Low	Low NK
IL7Rα deficiency	*IL7R*	AR	146661	Very low	Normal to high	Low	Normal NK
CD45 deficiency	*PTPRC*	AR	151460	Very low	Normal	Low	Normal γ/δ Τ cells
CD3δ deficiency	*CD3D*	AR	186790	Very low	Normal	Low	Normal NK, no γ/δ T cells
CD3ε deficiency	*CD3E*	AR	186830	Very low	Normal	Low	Normal NK, no γ/δ T cells
CD3ζ deficiency	*CD3Z*	AR	186780	Very low	Normal	Low	Normal NK, no γ/δ T cells
Coronin-1A deficiency	*CORO1A*	AR	605000	Very low	Normal	Low	Detectable thymus
LAT deficiency	*LAT*	AR	602354	Normal to low	Normal to low	High	Typical SCID or combined immunodeficiency, the latter with adenopathy, splenomegaly, recurrent infections, autoimmunity
2. T-B- SCID							
RAG deficiency	*RAG1* *RAG2*	AR	179615 179616	Very low	Very low	Decreased	Normal NK cell number, but increased risk of graft rejection, possibly due to activated NK cells
DCLRE1C (Artemis) deficiency	*DCLRE1C*	AR	605988	Very low	Very low	Decreased	Normal NK cell number, but increased risk of graft rejection, possibly due to activated NK cells, radiation sensitivity
DNA PKcs deficiency	*PRKDC*	AR	615966	Very low	Very low	Variable	Normal NK, radiation sensitivity, microcephaly
Cernunnos/XLF deficiency	*NHEJ1*	AR	611290	Very low	Very low	Decreased	Normal NK, radiation sensitivity, microcephaly
DNA ligase IV deficiency	*LIG4*	AR	601837	Very low	Very low	Decreased	Normal NK, radiation sensitivity, microcephaly
Adenosine deaminase (ADA) deficiency	*ADA*	AR	608958	Very low	Low, decreasing	Low, decreasing	Low NK, bone defects, may have pulmonary alveolar proteinosis, cognitive defects
AK2 defect	*AK2*	AR	103020	Very low	Very Low	Decreased	Reticular dysgenesis with neutropenia; deafness
Activated RAC2 defect	*RAC2*	AD GOF	602049	Very low	Very Low	Low, poor specific antibody responses	Recurrent bacterial and viral infections, lymphoproliferation; neutropenia
3. Combined immunodeficiency (CID), generally less profound than SCID							
CD40 ligand (CD154) deficiency	*CD40LG*	XL	308230	Normal to low	sIgM^+^IgD^+^ naïve B cells present; IgG^+^, IgA^+^, IgE^+^ memory B cells absent	IgM normal or high, other Ig isotypes low	Severe and opportunistic infections, idiopathic neutropenia; hepatitis and cholangitis, *Cryptosporidiu*m infections, cholangiocarcinoma; autoimmune blood cytopenias; peripheral neuroectodermal tumors
CD40 deficiency	*CD40*	AR	606843	Normal			Neutropenia, opportunistic infections, gastrointestinal and biliary tract and liver disease, *Cryptosporidium* infections
ICOS deficiency	*ICOS*	AR	604558	Normal	Normal	Low	Recurrent infections, autoimmunity, gastroenteritis, granulomas
ICOSL deficiency	*ICOSLG*	AR	605717	Low	Low	Low	Recurrent bacterial and viral infections, neutropenia
CD3γ deficiency	*CD3G*	AR	186740	Normal number, but low TCR expression	Normal	Normal	Immune deficiency and autoimmunity of variable severity
CD8 deficiency	*CD8A*	AR	186910	Absent CD8, Normal CD4	Normal	Normal	Recurrent infections, may be asymptomatic
ZAP-70 deficiency (ZAP70 LOF)	*ZAP70*	AR	269840	Low CD8 number, normal CD4 number but with poor function	Normal	Normal	May have immune dysregulation, autoimmunity
ZAP-70 combined hypomorphic and activating mutations	*ZAP70*	AR (LOF/GOF)	617006	Decreased CD8, normal or decreased CD4 cells	Normal or decreased	Normal IgA, low IgM, low/normal IgG; protective Ab responses to vaccines	Severe autoimmunity (bullous pemphigoid, inflammatory colitis
MHC class I deficiency	*TAP1*	AR	170260	Low CD8, normal CD4, absent MHC I on lymphocytes	Normal	Normal	Vasculitis, pyoderma gangrenosum
	*TAP2*	AR	170261				
	*TAPBP*	AR	601962				
	*B2M*	AR	109700				Sinopulmonary infections, cutaneous granulomas. Absent β2m associated proteins MHC-I, CD1a, CD1b, and CD1c
MHC class II deficiency group A, B, C, D	*CIITA*	AR	600005	Low CD4+ T cells, reduced MHC II expression on lymphocytes	Normal	Normal to low	Failure to thrive, respiratory and gastrointestinal infections, liver/biliary tract disease
	*RFXANK*	AR	603200				
	*RFX5*	AR	601863				
	*RFXAP*	AR	601861				
IKAROS deficiency	*IKZF1*	AD DN	603023	no memory T cells	no memory B cells	Low Ig,	recurrent sinopulmonary infections, pneumocystis early CID onset
DOCK8 deficiency	*DOCK8*	AR	243700	T cell lymphopenia, reduced naïve CD8 T cells, increased exhausted CD8+ T_EM_ cells, reduced MAIT, NKT cells, increased γδ T cells; poor proliferation; few Treg with poor function	increased total B cells, reduced memory B cells Poor peripheral B cell tolerance.	Low IgM, normal/high IgG and IgA, very high IgE, poor antibody responses	Low NK cells with poor function. Eosinophilia, recurrent infections, cutaneous viral, fungal and staphylococcal infections, severe atopy/allergic disease, cancer diathesis
DOCK2 deficiency	*DOCK2*	AR	603122	Low	Normal	IgG normal or low, poor antibody responses	Early invasive herpes viral, bacterial infections, Normal NK cell number, but defective function. Poor interferon responses in hematopoietic and non-hematopoietic cells
Polymerase and deficiency	*POLD1* *POLD2*	AR	174761 600815	Low CD4 T cells	Low B cells but normal maturation	Low igG	Recurrent respiratory tract infections, skin infections, warts and molluscum, short stature, intellectual disability
RHOH deficiency	*RHOH*	AR	602037	Normal, few naïve T cells, restricted repertoire, poor proliferation to CD3	Normal	Normal	HPV infection, lung granulomas, molluscum contagiosum, lymphoma
STK4 deficiency	*STK4*	AR	614868	CD4 lymphopenia, reduced naïve T cells, increased TEM and TEMRA cells, poor proliferation	Reduced memory B cells	Reduced IgM, increased IgG, IgA, IgE; impaired Ab responses	Intermittent neutropenia, bacterial, viral (HPV, EBV, molluscum), candidal infections, lymphoproliferation, autoimmune cytopenias, lymphoma, congenital heart disease
TCRα deficiency	*TRAC*	AR	615387	Absent TCRαβ except for a minor CD3-dim TCRαβ population; most T cells γδ; poor proliferation	Normal	Normal	Recurrent viral, bacterial, fungal infections, immune dysregulation and autoimmunity, diarrhea
LCK deficiency	*LCK*	AR	615758	Low CD4^+^, low Treg, restricted T cell repertoire, poor TCR signaling	Normal	Normal IgG and IgA, high IgM	Recurrent infections, immune dysregulation, autoimmunity
ITK deficiency	*ITK*	AR	186973	Progressive CD4 T cell lymphopenia; reduced T cell activation	Normal	Normal to low serum Ig	EBV associated B cell lymphoproliferation, lymphoma, immune dysregulation
MALT1 deficiency	*MALT1*	AR	615468	Normal number, poor proliferation	Normal	Normal levels, poor specific antibody response	Bacterial, fungal and viral infections
CARD11 deficiency	*CARD11*	AR LOF	615206	Normal number, predominantly naïve T cells, poor proliferation	Normal, transitional B cell predominance	Absent/low	*Pneumocystis jirovecii* pneumonia, bacterial and viral infections
BCL10 deficiency	*BCL10*	AR	616098	Normal number, few memory T and Treg cells, poor antigen and anti-CD3 proliferation	Normal number, decreased memory and switched B cells	Low	Recurrent bacterial and viral infections, candidiasis, gastroenteritis
IL-21 deficiency	*IL21*	AR	615767	Normal number, normal/low function	Low, decreased memory and switched B cells	Hypogammaglobulinemia, poor specific antibody responses; increased IgE	Severe early onset colitis, recurrent sinopulmonary infections
IL-21R deficiency	*IL21R*	AR	615207	Normal number, low cytokine production, poor antigen proliferation	Normal, decreased memory and switched B cells		Recurrent infections, *Pneumocystis jiroveci*, *Cryptosporidium* infections, liver disease
OX40 deficiency	*TNFRSF4*	AR	615593	Normal numbers, low antigen specific memory CD4+	Normal numbers, low memory B cells	Normal	Impaired immunity to HHV8, Kaposi’s sarcoma
IKBKB deficiency	*IKBKB*	AR	615592	Normal number, absent Treg and γ/δ T cells, impaired TCR activation	Normal number, poor function	Low	Recurrent bacterial, viral, fungal infections, opportunistic infections
NIK deficiency	*MAP 3 K14*	AR	604655	Normal number, poor proliferation to antigen	Low, low switched memory B cells	Low Ig’s	Low NK number and function, recurrent bacterial, viral and *Cryptosporidium* infections
RelB deficiency	*RELB*	AR	604758	Normal number, poor diversity, reduced proliferation to mitogens; no response to Ag	Marked increase in B cell number	Normal Ig levels but Impaired specific antibody responses	Recurrent infections
RelA haploinsufficiency	*RELA*	AD	618287	Normal/increased	Normal	Normal	Chronic mucocutaneous ulceration, Impaired NFkB activation; reduced production of inflammatory cytokines
Moesin deficiency	*MSN*	XL	300988	Normal number, defective migration, proliferation	Low number	Low Ig’s over time	Recurrent infections with bacteria, varicella, neutropenia
TFRC deficiency	*TFRC*	AR	616740	Normal number, poor proliferation	Normal number, low memory B cells	Low	Recurrent infections, neutropenia, thrombocytopenia
c-Rel deficiency	*REL*	AR	164910	Normal, decreased memory CD4, poor proliferation	Low, mostly naïve; few switched memory B cells, impaired proliferation	Low, poor specific antibody responses	Recurrent infections with bacteria, mycobacteria, salmonella and opportunistic organisms. Defective innate immunity
FCHO1 deficiency	*FCHO1*	AR	613437	Low, poor proliferation	Normal number	Normal	Recurrent infections (viral, mycobacteria, bacterial, fungal), lymphoproliferation, failure to thrive, increased activation-induced T cell death, defective clathrin-mediated endocytosis

SCID/CID spectrum: Infants with SCID who have maternal T cell engraftment may have T cells in normal numbers that do not function normally; these cells may cause autoimmune cytopenias or graft versus host disease. Hypomorphic mutations in several of the genes that cause SCID may result in Omenn syndrome (OS), or “leaky” SCID, or still less profound combined immunodeficiency (CID) phenotypes. Both OS and leaky SCID can be associated with > 300 autologous T cells/μL of peripheral blood and reduced, rather than absent, proliferative responses when compared with typical SCID caused by null mutations. A spectrum of clinical findings including typical SCID, OS, leaky SCID, CID, granulomas with T lymphopenia, autoimmunity and CD4 T lymphopenia can be found in an allelic series of *RAG1/2* and other SCID-associated genes. There can be clinical overlap between some genes listed here and those listed in Table 7

Total number of disorders in Table 1: 50

Total number of mutant genes: 58

New inborn errors of immunity: 8; New inborn errors of immunity: 8; *RAC2* GOF [18–21]; *ICOSLG* [22]; AD DN *IKZF1* [23]; *POLD1* [24, 25]; *POLD2* [24]; *RELA* [26, 27]; *REL* [28]; *FCHO1* [29]

*SCID* severe combined immunodeficiency, *CID* combined immunodeficiency, *EBV* Epstein-Barr virus, *MHC* major histocompatibility complex, *HPV* human papillomavirus, *Treg* T regulatory cell, *XL* X-linked inheritance, *AR* autosomal recessive inheritance, *AD* autosomal dominant inheritance, *LOF* loss-of-function, *GOF* gain-of-function

**Table 2:** Combined immunodeficiencies with associated or syndromic features

Disease	Genetic defect	Inheritance	OMIM	T cells	B cells	Ig	Associated features
1. Immunodeficiency with congenital thrombocytopenia							
Wiskott-Aldrich syndrome (WAS LOF)	*WAS*	XL	300392	Progressive decrease in numbers, abnormal lymphocyte responses to anti-CD3	Normal numbers	Low IgM and antibody responses to polysaccharides, often high IgA and IgE	Thrombocytopenia with small platelets, eczema, recurrent bacterial/viral infections, bloody diarrhea, lymphoma, autoimmune disease, IgA- nephropathy. Patients with XL-thrombocytopenia have later onset of complications and more favourable life expectancy but eventually develop similar complications as observed in WAS
WIP deficiency	*WIPF1*	AR	602357	Reduced, defective lymphocyte responses to anti-CD3	Normal or low	Normal, except for high IgE	Thrombocytopenia with or without small platelets, recurrent bacterial and viral infections, eczema, bloody diarrhea; WAS protein absent
Arp2/3-mediated filament branching defect	*ARPC1B*	AR	604223	Normal	Normal numbers	Normal except for high IgA and IgE	Mild thrombocytopenia with normal sized platelets, recurrent invasive infections; colitis, vasculitis, autoantibodies (ANA, ANCA), eosinophilia; defective Arp2/3 filament branching
2. DNA repair defects other than those listed in Table 1							
Ataxia-telangiectasia	*ATM*	AR	607585	Progressive decrease, poor proliferation to mitogens; may have low TRECs and T cells by newborn screening (NBS)	Normal	Often low IgA, IgE and IgG subclasses, increased IgM monomers; antibodies variably decreased	Ataxia, telangiectasia especially of sclerae; pulmonary infections; lymphoreticular and other malignancies; increased alpha fetoprotein; increased radiosensitivity, chromosomal instability and chromosomal translocations
Nijmegen breakage syndrome	*NBS1*	AR	602667	Progressive decrease; may have low TRECs and T cells by NBS	Variably reduced	Often low IgA, IgE, and IgG subclasses, increased IgM; antibodies variably decreased	Microcephaly, dysmorphic facies; lymphomas and solid tumors; increased radiosensitivity;, chromosomal instability
Bloom syndrome	*BLM*	AR	604610	Normal	Normal	Low	Short stature, dysmorphic facies sun-sensitive erythema; marrow failure; leukemia, lymphoma; chromosomal instability
Immunodeficiency with centromeric instability and facial anomalies (ICF types 1, 2, 3, 4)	*DNMT3B*	AR	602900	Decreased or normal, responses to PHA may be decreased	Decreased or normal	Hypogammaglobulinemia or agammaglobulinemia, variable antibody deficiency	Facial dysmorphic features, developmental delay, macroglossia; bacterial/opportunistic infections; malabsorption; cytopenias; malignancies; multiradial configurations of chromosomes 1, 9, 16
	*ZBTB24*	AR	614064	Decreased or normal			Facial dysmorphic features, macroglossia; bacterial/opportunistic infections; malabsorption; cytopenias; malignancies; multiradial configurations of chromosomes 1, 9, 16
	*CDCA7*	AR	609937	Decreased or normal; responses to PHA may be decreased			
	*HELLS*	AR	603946	Decreased or normal			
PMS2 deficiency	*PMS2*	AR	600259	Normal	Low B cells, switched and non-switched	Low IgG and IgA, high IgM, abnormal antibody responses	Recurrent infections; café-au-lait spots; lymphoma, colorectal carcinoma, brain tumors
RNF168 deficiency (Radiosensitivity, Immune Deficiency, Dysmorphic features, Learning difficulties [RIDDLE] syndrome)	*RNF168*	AR	612688	Normal	Normal	Low IgG or IgA	Short stature, mild defect of motor control to ataxia; normal intelligence to learning difficulties; mild facial dysmorphism to microcephaly; increased radiosensitivity
MCM4 deficiency	*MCM4*	AR	602638	Normal	Normal	Normal	NK cells: low number and function; viral infections (EBV, HSV, VZV); short stature; B cell lymphoma; adrenal failure
X-linked reticulate pigmentary disorder (POLA1 deficiency)	*POLA1*	XL	301220	Not assessed	Not assessed	Not assessed	Hyperpigmentation, characteristic facies, lung and GI involvement
POLE1 (Polymerase ε subunit 1) deficiency (FILS syndrome)	*POLE1*	AR	174762	Normal; decreased T cell proliferation	Low memory B cells	Low IgG2 and IgM, lack of antibody to PPS	Recurrent respiratory infections, meningitis; facial dysmorphism, livido, short stature
POLE2 (Polymerase ε subunit 2) deficiency	*POLE2*	AR	602670	Lymphopenia, lack of TRECS at NBS, absent proliferation in response to antigens	Very low	Hypogammaglobulinemia	Recurrent infections, disseminated BCG infections; autoimmunity (type 1 diabetes), hypothyroidism, facial dysmorphism
Ligase I deficiency	*LIG1*	AR	126391	Lymphopenia, increased γδ T cells, decreased mitogen response	Normal	Hypogammaglobulinemia, Reduced antibody responses	Recurrent bacterial and viral infections; growth retardation; sun sensitivity, radiation sensitivity; macrocytic red blood cells
NSMCE3 deficiency	*NSMCE3*	AR	608243	Decreased number, poor responses to mitogens and antigens	Normal	Normal IgG, IgA, normal to elevated IgM; decreased antibody responses to PPS	Severe lung disease (possibly viral); thymic hypoplasia; chromosomal breakage, radiation sensitivity
ERCC6L2 (Hebo deficiency)	*ERCC6L2*	AR	615667	Lymphopenia	Low	Normal	Facial dysmorphism, microcephaly; bone marrow failure
GINS1 deficiency	*GINS1*	AR	610608	Low or normal	Low or normal	High IgA, low IgM and IgG	Neutropenia; IUGR; NK cells very low
3. Thymic defects with additional congenital anomalies							
DiGeorge/velocardio-facial syndrome Chromosome 22q11.2 deletion syndrome (22q11.2DS)	*Large deletion (3 Mb) typically in chromosome 22 (TBX1)*	AD	602054	Decreased or normal, 5% have low TRECs at NBS and < 1500 CD3T cells/μL in neonatal period	Normal	Normal or decreased	Hypoparathyroidism; conotruncal cardiac malformation, velopalatal insufficiency; abnormal facies; intellectual disability
DiGeorge/velocardio-facial syndrome	Unknown	Sporadic		Decreased or normal			
TBX1 deficiency	*TBX1*	AD	602054	Decreased or normal, may have low TRECs at NBS			
CHARGE syndrome	*CHD7*	AD	608892	Decreased or normal, may have low TRECs at NBS; response to PHA may be decreased	Normal	Normal or decreased	Coloboma of eye; heart anomaly; choanal atresia; intellectual disability; genital and ear anomalies, CNS malformation; some are SCID-like
	*SEMA3E*	AD	608166				
	Unknown						
Winged helix nude FOXN1 deficiency	*FOXN1*	AR	601705	Very low	Normal	Decreased	Severe infections; abnormal thymic epithelium, immunodeficiency; congenital alopecia, nail dystrophy; neural tube defect
FOXN1 haploinsufficiency	*FOXN1*	AD	600838	Severe T cell lymphopenia at birth, normalised by adulthood	Normal/low	Not assessed	Recurrent, viral and bacterial respiratory tract infections; skin involvement (eczema, dermatitis), nail dystrophy
Chromosome 10p13-p14 deletion syndrome (10p13-p14DS)	*Del10p13-p14*	AD	601362	Normal, rarely lymphopenia and decreased lymphoproliferation to mitogens and antigens; hypoplastic thymus may be present	Normal	Normal	Hypoparathyroidism; renal disease; deafness; growth retardation; facial dysmorphism; cardiac defects may be present; recurrent infections ±
Chromosome 11q deletion syndrome (Jacobsen syndrome)	*11q23del*	AD	147791	Lymphopenia; low NK cells	Decreased B cells and switched memory B cells	Hypogammaglobulinemia, decreased antibody responses	Recurrent respiratory infections; multiple warts; facial dysmorphism, growth retardation
4. Immuno-osseous dysplasias							
Cartilage hair hypoplasia (CHH)	*RMRP*	AR	157660	Varies from severely decreased (SCID) to normal; impaired lymphocyte proliferation	Normal	Normal or reduced, antibodies variably decreased	Short-limbed dwarfism with metaphyseal dysostosis; sparse hair; bone marrow failure; autoimmunity; susceptibility to lymphoma and other cancers; impaired spermatogenesis; neuronal dysplasia of the intestine
Schimke immuno-osseous dysplasia	*SMARCAL1*	AR	606622	Decreased	Normal	Normal	Short stature, spondiloepiphyseal dysplasia, intrauterine growth retardation; nephropathy; bacterial, viral, fungal infections; may present as SCID; bone marrow failure
MYSM1 deficiency	*MYSM1*	AR	612176	T cell lymphopenia, reduced naïve T cells, low NK cells	B cell deficiency	Hypogammaglobulinemia	Short stature; recurrent infections; congenital bone marrow failure, myelodysplasia; immunodeficiency affecting B cells and granulocytes; skeletal anomalies; cataracts; developmental delay
MOPD1 deficiency (Roifman syndrome)	*RNU4ATAC*	AR	601428	Decreased NK cell function	Decreased total and memory B cells	Hypogammaglobulinemia, variably decreased specific antibodies	Recurrent bacterial infections; lymphadenopathy; spondyloepiphyseal dysplasia, extreme intrauterine growth retardation; retinal dystrophy; facial dysmorphism; may present with microcephaly; short stature
Immunoskeletal dysplasia with neurodevelopmental abnormalities (EXTL3 deficiency)	*EXTL3*	AR	617425	Decreased	Normal	Decreased to normal	Short stature; cervical spinal stenosis, neurodevelopmental impairment; eosinophilia; may have early infant mortality
5. Hyper IgE syndromes (HIES)							
AD-HIES STAT3 deficiency (Job syndrome)	*STAT3*	AD LOF (dominant negative)	147060	Normal overall; Th17, T follicular helper, MAIT, NKT cells decreased, Tregs may be increased; impaired responses to STAT3-activatng cytokines	Normal, reduced memory B cells, BAFF expression increased, impaired responses to STAT3-activating cytokines	Very high IgE, specific antibody production decreased	Distinctive facial features (broad nasal bridge); bacterial infections (boils, pulmonary abscesses, pneumatoceles) due to *S. aureus*, pulmonary aspergillus, *Pneumocystis jirovecii*; eczema, mucocutaneous candidiasis; hyperextensible joints, osteoporosis and bone fractures, scoliosis, retained primary teeth; coronary and cerebral aneurysms
IL6 receptor deficiency	*IL6R*	AR	147880	Normal/increased; normal responses to mitogens	Normal total and memory B; reduced switched memory B	Normal/low serum IgM, G, A. Very high IgE; specific antibody production low	Recurrent pyogenic infections, cold abscesses; high circulating IL-6 levels
IL6 signal transducer (IL6ST) deficiency	*IL6ST*	AR	618523	Decreased Th17 cells	Reduced switched and non-switched memory B cells	High IgE, specific antibody production variably affected	Bacterial infections, boils, eczema, pulmonary abscesses, pneumatoceles; bone fractures; scoliosis; retention of primary teeth; craniosynostosis
ZNF341 deficiency AR-HIES	*ZNF341*	AR	618282	Decreased Th17 and NK cells	Normal, reduced memory B cells, impaired responses to STAT3-activaitng cytokines	High IgE and IgG, specific antibody production decreased	Phenocopy of AD-HIES; mild facial dysmorphism; early onset eczema, MCC, bacterial skin infections, abscesses, recurrent bacterial respiratory infections (S. aureus), lung abscesses and pneumatoceles; hyperextensible joints; bone fractures and retention of primary teeth
ERBIN deficiency	*ERBB2IP*	AD	606944	Increased circulating Treg	Normal	Moderately increased IgE	Recurrent respiratory infections, susceptibility to S. aureus, eczema; hyperextensible joints, scoliosis; arterial dilatation in some patients
Loeys-Dietz syndrome (TGFBR deficiency)	*TGFBR1*	AD	609192	Normal	Normal	Elevated IgE	Recurrent respiratory infectons; eczema, food allergies; hyper-extensible joints, scoliosis, retention of primary teeths; aortic aneurisms.
	*TGFBR2*		610168				
Comel-Netherton syndrome	*SPINK5*	AR	605010	Normal	Low switched and non-switched B cells	High IgE and IgA, Antibody variably decreased	Congenital ichthyosis, bamboo hair, atopic diathesis; increased bacterial infections; failure to thrive
PGM3 deficiency	*PGM3*	AR	172100	CD8 and CD4 T cells may be decreased	Low B and memory B cells	Normal or elevated IgG and IgA, most with high IgE, eosinophilia	Severe atopy; autoimmunity; bacterial and viral infections; skeletal anomalies/dysplasia: short stature, brachydactyly, dysmorphic facial features; intellectual disability and cognitive impairment, delayed CNS myelination in some affected individuals
CARD11 deficiency (heterozygous DN)	*CARD11*	AD LOF	617638	Normal overall, but defective T cell activation and proliferation; skewing toward Th2	Normal to low	High IgE, poor specific antibody production; impaired activation of both NF-κB and mTORC1 pathways	Variable atopy, eczema, food allergies, eosinophilia; cutaneous viral infections, recurrent respiratory infections; lymphoma; CID
6. Defects of vitamin B12 and folate metabolism							
Transcobalamin 2 deficiency	*TCN2*	AR	613441	Normal	Variable	Decreased	Megaloblastic anemia, pancytopenia; if untreated (B12) for prolonged periods results in intellectual disability
SLC46A1/PCFT deficiency causing hereditary folate malabsorption	*SLC46A1*	AR	229050	Variable numbers and activation profile	Variable	Decreased	Megaloblastic anemia, failure to thrive; if untreated for prolonged periods results in intellectual disability
Methylene-tetrahydrofolate dehydrogenase 1 (MTHFD1) deficiency	*MTHFD1*	AR	172460	Low thymic output, normal in vitro proliferation	Low	Decreased/poor antibody responses to conjugated polysaccharide antigens	Recurrent bacterial infection, *Pneumocystis jirovecii;* megaloblastic anemia; failure to thrive; neutropenia; seizures, intellectual disability; folate-responsive
7. Anhidrotic ectodermodysplasia with immunodeficiency (EDA-ID)							
EDA-ID due to NEMO/IKBKG deficiency (ectodermal dysplasia, immune deficiency)	*IKBKG*	XL	300248	Normal or decreased, TCR activation impaired	Normal; Low memory and isotype switched B cells	Decreased, some with elevated IgA, IgM, poor specific antibody responses, absent antibodies to polysaccharide antigens	Anhidrotic ectodermal dysplasia (in some); various infections (bacteria, mycobacteria, viruses, fungi); colitis; conical teeth, variable defects of skin, hair and teeth; monocyte dysfunction
EDA-ID due to IKBA GOF mutation	*NFKBIA*	AD GOF	164008	Normal total T cells, TCR activation impaired	Normal B cell numbers, impaired BCR activation, low memory and isotype switched B cells	Decreased IgG and IgA, elevated IgM, poor specific antibody responses, absent antibody to polysaccharide antigens	Anhidrotic ectodermal dysplasia; various infections (bacteria, mycobacteria, viruses, fungi); colitis; variable defects of skin, hair and teeth; T cell and monocyte dysfunction
EDA-ID due to IKBKB GOF mutation	*IKBKB*	AD GOF	618204	Decreased T cells, impaired TCR activation	Normal number, poor function	Reduced	Recurrent bacterial, viral, fungal infections; variable ectodermal defects
8. Calcium channel defects							
ORAI-1 deficiency	*ORAI1*	AR	610277	Normal, defective TCR mediated activation	Normal	Normal	Autoimmunity; EDA; non-progressive myopathy
STIM1 deficiency	*STIM1*	AR	605921				
9. Other defects							
Purine nucleoside phosphorylase (PNP) deficiency	*PNP*	AR	164050	Progressive decrease	Normal	Normal or low	Autoimmune hemolytic anemia; neurological impairment
Immunodeficiency with multiple intestinal atresias	*TTC7A*	AR	609332	Variable, but sometimes absent or low TRECs at NBS; may have SCID phenotype at birth	Normal or low	Markedly decreased IgG, IgM, IgA	Bacterial (sepsis), fungal, viral infections; multiple intestinal atresias, often with intrauterine polyhydramnios and early demise
Tricho-Hepato-Enteric Syndrome (THES)	*TTC37*	AR	222470	Impaired IFNγ production	Variably low numbers of switched memory B cells	Hypogammaglobulinemia, may have low antibody responses	Respiratory infections; IUGR; facial dysmorphic features, wooly hair; early onset intractable diarrhea, liver cirrhosis; platelet abnormalities
	*SKIV2L*		614602				
Hepatic veno-occlusive disease with immunodeficiency (VODI)	*SP110*	AR	604457	Normal (decreased memory T cells)	Normal (decreased memory B cells)	Decreased IgG, IgA, IgM, absent germinal center and tissue plasma cells	Hepatic veno-occlusive disease; susceptibility to *Pneumocystis jirovecii* pneumonia, CMV, candida; thrombocytopenia; hepatosplenomegaly; cerebrospinal leukodystrophy
BCL11B deficiency	*BCL11B*	AD	617237	Low, poor proliferation	Normal	Normal	Congenital abnormalities, neonatal teeth, dysmorphic facies; absent corpus callosum, neurocognitive deficits
EPG5 deficiency (Vici syndrome)	*EPG5*	AR	615068	Profound depletion of CD4+ cells	Defective	Decreased (particularly IgG2)	Agenesis of the corpus callosum; cataracts; cardiomyopathy; skin hypopigmentation; intellectual disability; microcephaly; recurrent infections, chronic mucocutaneous candidiasis
HOIL1 deficiency	*RBCK1*	AR	610924	Normal numbers	Normal, decreased memory B cells	Poor antibody responses to polysaccharides	Bacterial infections; autoinflammation; amylopectinosis
HOIP deficiency	*RNF31*	AR	612487	Normal numbers	Normal, decreased memory B cells	decreased	Bacterial infections; autoinflammation; amylopectinosis; lymphangiectasia
Hennekam-lymphangiectasia-lymphedema syndrome	*CCBE1*	AR	612753	Low/variable	Low/variable	decreased	Lymphangiectasia and lymphedema with facial abnormalities and other dysmorphic features
	*FAT4*	AR	612411	Low/variable	Low/variable	decreased	Lymphangiectasia and lymphedema with facial abnormalities and other dysmorphic features
Activating de novo mutations in nuclear factor, erythroid 2- like (NFE2L2)	*NFE2L2*	AD	617744	Not reported	Decreased switched memory B cells	Hypogammaglobulinemia, decreased antibody responses	Recurrent respiratory and skin infections; growth retardation, developmental delay; white matter cerebral lesions; increased level of homocysteine; increased expression of stress response genes
STAT5b deficiency	*STAT5B*	AR	245590	Modestly decreased, reduced Treg number and function	Normal	hypergammaglobulinemia, increased IgE	Growth-hormone insensitive dwarfism; dysmorphic features; eczema; lymphocytic interstitial pneumonitis; prominent autoimmunity
STAT5b deficiency	*STAT5B*	AD (dominant negative)	604260	Normal	Normal	Increased IgE	Growth-failure; eczema (no immune defects compared to AR STAT5 deficiency)
Kabuki syndrome (type 1 and 2)	*KMT2D*	AD	602113	Normal	Normal	Low IgA and occasionally low IgG	Typical facial abnormalities, cleft or high arched palate, skeletal abnormalities, short stature; intellectual disability; congenital heart defects; recurrent infections (otitis media, pneumonia) in 50% of patients; autoimmunity may be present
	*KDM6A*	XL (females may be affected)	300128				
KMT2A deficiency (Wiedemann-Steiner syndrome)	*KMT2A*	AD	605130	Normal	Decreased switched and non-switched memory B cells	Hypogammaglobulinemia, decreased antibody responses	Respiratory infections; short stature; hypertelorism; hairy elbows; developmental delay, intellectual disability

Total number of disorders in Table 2: 58

Total number of mutant genes in Table 2: 62

New inborn errors of immunity: 12; *LIG1* [30]; *FOXN1* haploinsufficiency [31]; *IL6R* [32, 33]; *IL6ST* [34, 35]; *ZNF341* [36, 37]; *ERBB2IP* [38]; *TGFBR1* [39]; *TGFBR2* [39]; AD LOF *CARD11* [40, 41]; AD GOF *IKBKB* [42]; *SKIV2L* [43]; *NFE2L2* [44]

Unknown cause of DiGeorge syndrome, unknown cause of CHARGE syndrome, unknown gene(s) within 10p13–14 deletion responsible for phenotype

*EDA* ectodermal dysplasia anhydrotic, *HSV* herpes simplex virus, *VZV* varicella zoster virus, *BCG* Bacillus Calmette-Guerin, *NBS* newborn screen, *TREC* T cell receptor excision circle (biomarker for low T cells used in NBS), *IUGR* interuterine growth retardation

**Table 3:** Predominantly antibody deficiencies

Disease	Genetic defect	Inheritance	OMIM	Ig	Associated features
1. Severe reduction in all serum immunoglobulin isotypes with profoundly decreased or absent B cells, agammaglobulinemia					
BTK deficiency, X-linked agammaglobulinemia (XLA)	*BTK*	XL	300300	All isotypes decreased in majority of patients, some patients have detectable immunoglobulins	Severe bacterial infections, normal numbers of pro-B cells
μ heavy chain deficiency	*IGHM*	AR	147020	All isotypes decreased	Severe bacterial infections, normal numbers of pro-B cells
λ5 deficiency	*IGLL1*	AR	146770		
Igα deficiency	*CD79A*	AR	112205		
Igβ deficiency	*CD79B*	AR	147245		
BLNK deficiency	*BLNK*	AR	604515		
p110δ deficiency	*PIK3CD*	AR	602839		Severe bacterial infections; autoimmune complications (IBD)
p85 deficiency	*PIK3R1*	AR	615214		Severe bacterial infections, cytopenias, decreased or absent pro-B cells
E47 transcription factor deficiency	*TCF3*	AD	616941		Recurrent bacterial infections
	*TCF3*	AR	147141		Severe, recurrent bacterial infections, failure to thrive
SLC39A7 (ZIP7) deficiency	*SLC39A7*	AR	601416		Early onset infections, blistering dermatosis, failure to thrive, thrombocytopenia
Hoffman syndrome/TOP2B deficiency	*TOP2B*	AD	126431		Recurrent infections, facial dysmorphism, limb anomalies
2. Severe reduction in at least 2 serum immunoglobulin isotypes with normal or low number of B cells, CVID phenotype					
Common variable immune deficiency with no gene defect specified (CVID)	Unknown	Variable		Low IgG and IgA and/or IgM	Clinical phenotypes vary: most have recurrent infections, some have polyclonal lymphoproliferation, autoimmune cytopenias and/or granulomatous disease
Activated p110δ syndrome (APDS)	*PIK3CD* GOF	AD	615513 (APDS1)	Normal/increased IgM, reduced IgG and IgA	Severe bacterial infections; reduced memory B cells and increased transitional B cells, EBV ± CMV viremia, lymphadenopathy/splenomegaly, autoimmunity, lymphoproliferation, lymphoma
	*PIK3R1*	AD	616005 (APDS2)		Severe bacterial infections, reduced memory B cells and increased transitional B cells, lymphadenopathy/splenomegaly, lymphoproliferation, lymphoma; developmental delay
PTEN deficiency (LOF)	*PTEN*	AD	158350	Normal/Decreased	Recurrent infections, Lymphoproliferation, Autoimmunity; developmental delay
CD19 deficiency	*CD19*	AR	107265	Low IgG and IgA and/or IgM	Recurrent infections, may have glomerulonephritis (CD81 mutation abolishes expression of CD19, thereby phenocopying CD19 mutations)
CD81 deficiency	*CD81*	AR	186845	Low IgG, low or normal IgA and IgM	
CD20 deficiency	*CD20*	AR	112210	Low IgG, normal or elevated IgM and IgA	Recurrent infections
CD21 deficiency	*CD21*	AR	120650	Low IgG, impaired anti-pneumococcal response	Recurrent infections
TACI deficiency^#^	*TNFRSF13B*	AR or AD	604907	Low IgG and IgA and/or IgM	Variable clinical expression and penetrance for monoallelic variants
BAFF receptor deficiency	*TNFRSF13C*	AR	606269	Low IgG and IgM,	Variable clinical expression
TWEAK deficiency	*TNFSF12*	AD	602695	Low IgM and A, lack of anti-pneumococcal antibody	Pneumonia, bacterial infections, warts, thrombocytopenia. Neutropenia
TRNT1 deficiency	*TRNT1*	AR	612907	B cell deficiency and hypogammaglobulinemia	congenital sideroblastic anemia, deafness, developmental delay
NFKB1 deficiency	*NFKB1*	AD	164011	Normal or low IgG, IgA, IgM, low or normal B cells, low memory B cells	Recurrent sinopulmonary infections, COPD, EBV proliferation, autoimmune cytopenias, alopecia and autoimmune thyroiditis
NFKB2 deficiency	*NFKB2*	AD	615577	Low serum IgG, A and M; low B cell numbers	Recurrent sinopulmonary infections, alopecia and endocrinopathies
IKAROS deficiency	*IKZF1*	AD (haploinsufficiency)	603023	Low IgG, IgA, IgM, low or normal B cells; B cells and Ig levels reduce with age	Decreased pro-B cells, recurrent sinopulmonary infections; increased risk of ALL, autoimmunity, CVID phenotype
IRF2BP2 deficiency	*IRF2BP2*	AD	615332	Hypogammaglobulinemia, absent IgA	Recurrent infections, possible autoimmunity and inflammatory disease
ATP6AP1 deficiency	*ATP6AP1*	XL	300972	Variable immunoglobulin findings	Hepatopathy, leukopenia, low copper
ARHGEF1 deficiency	*ARHGEF1*	AR	618459	Hypogammaglobulinemia; lack of antibody	Recurrent infections, bronchiectasis
SH3KBP1 (CIN85) deficiency	*SH3KBP1*	XL	300310	IgM, IgG deficiency; loss of antibody	Severe bacterial infections
SEC61A1 deficiency	*SEC61A1*	AD	609213	Hypogammaglobulinemia	Severe recurrent respiratory tract infections
RAC2 deficiency	*RAC2*	AR	602049	Low IgG, IgA, IgM, low or normal B cells; reduced Ab responses following vaccination	Recurrent sinopulmonary infections, selective IgA deficiency; poststreptococcal glomerulonephritis; urticaria
Mannosyl-oligosaccharide glucosidase deficiency	*MOGS*	AR	601336	Low IgG, IgA, IgM, increased B cells; poor Ab responses following vaccination	Bacterial and viral infections; severe neurologic disease; also known as congenital disorder of glycosylation type IIb (CDG-IIb)
3. Severe reduction in serum IgG and IgA with normal/elevated IgM and normal numbers of B cells, hyper IgM					
AID deficiency	*AICDA*	AR	6055258	IgG and IgA decreased, IgM increased; normal memory B cells but lacking somatic hypermutation	Bacterial infections, enlarged lymph nodes and germinal centers; autoimmunity
		AD	605257	IgG absent or decreased, IgA undetected, IgM increased; normal memory B cells with intact somatic hypermutation	Bacterial infections, enlarged lymph nodes and germinal centers. Mutations uniquely localize to the nuclear export signal.
UNG deficiency	*UNG*	AR	191525	IgG and IgA decreased, IgM increased	Enlarged lymph nodes and germinal centers
INO80 deficiency	*INO80*	AR	610169	IgG and IgA decreased, IgM increased	Severe bacterial infections
MSH6 deficiency	*MSH6*	AR	600678	Variable IgG, defects, increased IgM in some, normal B cells, low switched memory B cells, Ig class switch recombination and somatic hypermutation defects	Family or personal history of cancer
4. Isotype, light chain, or functional deficiencies with generally normal numbers of B cells					
Ig heavy chain mutations and deletions	Mutation or chromosomal deletion at 14q32	AR		One or more IgG and/or IgA subclasses as well as IgE may be absent	May be asymptomatic
Kappa chain deficiency	*IGKC*	AR	147200	All immunoglobulins have lambda light chain	Asymptomatic
Isolated IgG subclass deficiency	Unknown	?		Reduction in one or more IgG subclass	Usually asymptomatic, a minority may have poor antibody response to specific antigens and recurrent viral/bacterial infections
IgG subclass deficiency with IgA deficiency	Unknown	?		Reduced IgA with decrease in one or more IgG subclass	Recurrent bacterial infections
May be asymptomatic					
Selective IgA deficiency	Unknown	?		Absent IgA with other isotypes normal, normal subclasses and specific antibodies	May be asymptomatic Bacterial infections, autoimmunity mildly increased
Specific antibody deficiency with normal Ig levels and normal B cells	Unknown	?		Normal	Reduced ability to produce antibodies to specific antigens
Transient hypogammaglobulinemia of infancy	Unknown	?		IgG and IgA decreased	Normal ability to produce antibodies to vaccine antigens, usually not associated with significant infections
CARD11 GOF	*CARD11*	AD GOF	616452	Polyclonal B cell lymphocytosis due to constitutive NF-κB activation	Splenomegaly, lymphadenopathy, poor vaccine response
Selective IgM deficiency	Unknown	?		Absent serum IgM	Pneumococcal/bacterial

Common variable immunodeficiency disorders (CVID) include several clinical and laboratory phenotypes that may be caused by distinct genetic and/or environmental factors. Some patients with CVID and no known genetic defect have markedly reduced numbers of B cells as well as hypogammaglobulinemia. Identification of causal variants can assist in defining treatment. In addition to monogenic causes on this table, a small minority of patients with XLP (Table 4), WHIM syndrome (Table 6), ICF (Table 2), VODI (Table 2), thymoma with immunodeficiency (Good syndrome), or myelodysplasia are first seen by an immunologist because of recurrent infections, hypogammaglobulinemia, and normal or reduced numbers of B cells

Total number of disorders in Table 3: 46

Total number of mutant genes in Table 3: 39

New disorders: 9: AR *PIK3CD* [46–48]; AR *TCF3* [49, 50]; *SLC39A7* [51]; *TOP2B* [52]; *ARHGEF1* [53]; *SH3KBP1* [54]; *SEC61A1* [55]; *AR LOF RAC2* [56]; AD *AICDA*

*EBV* Epstein-Barr virus, *COPD* chronic obstructive pulmonary disease

^#^Heterozygous variants in *TNFRSF13B* have been detected in healthy individuals, thus such variants are likely to be disease-modifying rather than disease-causing

**Table 4:** Diseases of immune dysregulation

Disease	Genetic defect	Inheritance	OMIM	Circulating T cells	Circulating B cells	Functional defect	Associated features
1. Familial hemophagocytic lymphohistiocytosis (FHL syndromes)							
Perforin deficiency (FHL2)	*PRF1*	AR	170280	Increased activated T cells	Normal	Decreased to absent NK and CTL activities cytotoxicity	Fever, HSM, hemophagocytic lymphohistiocytosis (HLH), cytopenias
UNC13D/Munc13–4 deficiency (FHL3)	*UNC13D*	AR	608897	Increased activated T cells	Normal	Decreased to absent NK and CTL activities (cytotoxicity and/or degranulation)	Fever, HSM, HLH, cytopenias,
Syntaxin 11 deficiency (FHL4)	*STX11*	AR	605014				
STXBP2/Munc18–2 deficiency (FHL5)	*STXBP2*	AR or AD	601717				
FAAP24 deficiency	*FAAP24*	AR	610884	Increased activated T cells	Normal	Failure to kill autologous EBV transformed B cells. Normal NK cell function	EBV-driven lymphoproliferative disease
SLC7A7 deficiency	*SLC7A7*	AR	222700	Normal	Normal	Hyper-inflammatory response of macrophages Normal NK cell function	Lysinuric protein intolerance, bleeding tendency, alveolar proteinosis
2. FHL syndromes with hypopigmentation							
Chediak-Higashi syndrome	*LYST*	AR	606897	Increased activated T cells	Normal	Decreased NK and CTL activities (cytotoxicity and/or degranulation)	Partial albinism, recurrent infections, fever, HSM, HLH, giant lysosomes, neutropenia, cytopenias, bleeding tendency, progressive neurological dysfunction
Griscelli syndrome, type 2	*RAB27A*	AR	603868	Normal	Normal	Decreased NK and CTL activities (cytotoxicity and/or degranulation)	Partial albinism, fever, HSM, HLH, cytopenias
Hermansky-Pudlak syndrome, type 2	*AP3B1*	AR	603401	Normal	Normal	Decreased NK and CTL activities (cytotoxicity and/or degranulation)	Partial albinism, recurrent infections, pulmonary fibrosis, increased bleeding, neutropenia, HLH
Hermansky-Pudlak syndrome, type 10	*AP3D1*	AR	617050	Normal	Normal	Decreased NK and CTL activities (cytotoxicity and/or degranulation)	Oculocutaneous albinism, severe neutropenia, recurrent infections, seizures, hearing loss and neurodevelopmental delay
3. Regulatory T cell defects							
IPEX, immune dysregulation, polyendocrinopathy, enteropathy X-linked	*FOXP3*	XL	300292	Normal	Normal	Lack of (and/or impaired function of) CD4^+^ CD25^+^ FOXP3^+^ regulatory T cells (Tregs)	Autoimmune enteropathy, early onset diabetes, thyroiditis hemolytic anemia, thrombocytopenia, eczema, elevated IgE and IgA
CD25 deficiency	*IL2RA*	AR	147730	Normal to decreased	Normal	No CD4 + C25+ cells with impaired function of Tregs cells	Lymphoproliferation, autoimmunity, impaired T cell proliferation in vitro
CD122 deficiency	*IL2RB*	AR	618495	Increased memory CD8 T cells, decreased Tregs	Increased memory B cells	Diminished IL2Rβ expression, dysregulated signaling in response to IL-2/IL-15; increased immature NK cells	Lymphoproliferation, lymphadenopathy, hepatosplenomegaly, autoimmune hemolytic anemia, dermatitis, enteropathy, hypergammaglobulinemia, recurrent viral (EBV, CMV) infections
CTLA4 haploinsufficiency (ALPS-V)	*CTLA4*	AD	123890	Decreased	Decreased	Impaired function of Tregs.	Autoimmune cytopenias, enteropathy, interstitial lung disease, extra-lymphoid lymphocytic infiltration, recurrent infections
LRBA deficiency	*LRBA*	AR	606453	Normal or decreased CD4 numbers T cell dysregulation	Low or normal numbers of B cells	Reduced IgG and IgA in most	Recurrent infections, inflammatory bowel disease, autoimmunity
DEF6 deficiency	*DEF6*	AR	610094	Mild CD4 and CD8 lymphopenia	Low or normal numbers of B cells	Impaired Treg function	Enteropathy, hepatosplenomegaly, cardiomyopathy, recurrent infections
STAT3 GOF mutation	*STAT3*	AD GOF	102582	Decreased	Decreased	Enhanced STAT3 signaling, leading to increased Th17 cell differentiation, lymphoproliferation and autoimmunity. Decreased Tregs and impaired function	Lymphoproliferation, solid organ autoimmunity, recurrent infections
BACH2 deficiency	*BACH2*	AD	605394	Progressive T cell lymphopenia	Impaired memory B cell development	Haploinsufficiency for a critical lineage specification transcription factor	Lymphocytic colitis, sinopulmonary infections
FERMT1 deficiency	*FERMT1*	AR	173650	Normal	Normal	Intracellular accumulation of IgG, IgM, IgA, and C3 in colloid bodies under the basement membrane	Dermatosis characterized by congenital blistering, skin atrophy, photosensitivity, skin fragility, and scaling
4. Autoimmunity with or without lymphoproliferation							
APECED (APS-1), autoimmune polyendocrinopathy with candidiasis and ectodermal dystrophy	*AIRE*	AR or AD	240300	Normal	Normal	AIRE serves as check-point in the thymus for negative selection of autoreactive T cells and for generation of Tregs	Autoimmunity: hypoparathyroidism, hypothyroidism, adrenal insufficiency, diabetes, gonadal dysfunction and other endocrine abnormalities; dental enamel hypoplasia, alopecia areata enteropathy, pernicious anemia; chronic mucocutaneous candidiasis
ITCH deficiency	*ITCH*	AR	606409	Not assessed	Not assessed	Itch deficiency may cause immune dysregulation by affecting both anergy induction in auto-reactive effector T cells and generation of Tregs	Early-onset chronic lung disease (interstitial pneumonitis), autoimmunity (thyroiditis, type I diabetes, chronic diarrhea/enteropathy, and hepatitis), failure to thrive, developmental delay, dysmorphic facial features
Tripeptidyl-peptidase II deficiency	*TPP2*	AR	190470	Decreased	Decreased	TPP2 deficiency results in premature immunosenescence and immune dysregulation	Variable lymphoproliferation, severe autoimmune cytopenias, hypergammaglobulinemia, recurrent infections
JAK1 GOF	*JAK1*	AD GOF	147795	Not assessed	Not assessed	Hyperactive JAK1	HSM, eosinophilia, eosinophilic enteritis, thyroid disease, poor growth, viral infections
Prolidase deficiency	*PEPD*	AR	613230	Normal	Normal	Peptidase D	Autoantibodies common, chronic skin ulcers, eczema, infections
5. Immune dysregulation with colitis							
IL-10 deficiency	*IL10*	AR	124092	Normal	Normal	No functional IL-10 secretion	Inflammatory bowel disease (IBD), folliculitis, recurrent respiratory diseases, arthritis,
IL-10R deficiency	*IL10RA*	AR	146933	Normal	Normal	Leukocytes unresponsive to IL-10	IBD, folliculitis, recurrent respiratory diseases, arthritis, lymphoma
	*IL10RB*	AR	123889	Normal	Normal	Leukocytes unresponsive to IL-10, and IL-22, IL-26, IL-28A, IL-28B and IL-29	
NFAT5 haploinsufficiency	*NFAT5*	AD	604708	Normal	Normal	Decreased memory B cells and plasmablasts	IBD, recurrent sinopulmonary infections
TGFB1 deficiency	*TGFB1*	AR	618213	Normal	Normal	Decreased T cell proliferation in response to anti-CD3	IBD, immunodeficiency, recurrent viral infections, microcephaly, and encephalopathy
RIPK1	*RIPK1*	AR	618108	Reduced	Normal/reduced	Reduced activation of MAPK, NFkB pathways to	Recurrent infections, early-onset IBD, progressive polyarthritis
6. Autoimmune lymphoproliferative syndrome (ALPS, Canale-Smith syndrome)							
ALPS-FAS	*TNFRSF6*	AD AR	134637	Increased TCR α/β+CD4^−^CD8^−^ double negative (DN) T cells	Normal, low memory B cells	Apoptosis defect FAS mediated	Splenomegaly, adenopathies, autoimmune cytopenias, increased lymphoma risk, IgG and A normal or increased, elevated serum FasL, IL-10, vitamin B12
ALPS-FASLG	*TNFSF6*	AR	134638	Increased DN T cells	Normal	Apoptosis defect FASL mediated	Splenomegaly, adenopathies, autoimmune cytopenias, SLE, soluble FasL is not elevated
ALPS-Caspase10	*CASP10*	AD	601762	Increased DN T cells	Normal	Defective lymphocyte apoptosis	Adenopathies, splenomegaly, autoimmunity
ALPS-Caspase 8	*CASP8*	AR	601763	Slightly increased DN T cells	Normal	Defective lymphocyte apoptosis and activation	Adenopathies, splenomegaly, bacterial and viral infections, hypogammaglobulinemia
FADD deficiency	*FADD*	AR	602457	Increased DN T cells	Normal	Defective lymphocyte apoptosis	Functional hyposplenism, bacterial and viral infections, recurrent episodes of encephalopathy and liver dysfunction
7. Susceptibility to EBV and lymphoproliferative conditions							
SAP deficiency (XLP1)	*SH2D1A*	XL	300490	Normal or Increased activated T cells	Reduced Memory B cells	Reduced NK cell and CTL cytotoxic activity	Clinical and immunologic features triggered by EBV infection: HLH, Lymphoproliferation, Aplastic anemia, Lymphoma. Hypogammaglobulinemia, Absent iNKT cells
XIAP deficiency (XLP2)	*XIAP*	XL	300079	Normal or Increased activated T cells; low/normal iNK T cells	Normal or reduced Memory B cells	Increased T cells susceptibility to apoptosis to CD95 and enhanced activation-induced cell death (AICD)	EBV infection, Splenomegaly, lymphoproliferation HLH, Colitis, IBD, hepatitis Low iNKT cells
CD27 deficiency	*CD27*	AR	615122	Normal	No memory B cells	hypogammaglobulinemia; poor Ab responses to some vaccines/infections	Features triggered by EBV infection, HLH, aplastic anemia, low iNKT cells, B-lymphoma
CD70 deficiency	*CD70*	AR	602840	Normal number, low Treg, poor activation and function	Decreased memory B cells	hypogammaglobulinemia; poor Ab responses to some vaccines/infections	EBV susceptibility, Hodgkin lymphoma; autoimmunity in some patients
CTPS1 deficiency	*CTPS1*	AR	615897	Normal to low, but reduced activation, proliferation	Decreased memory B cells	Normal/high IgG poor proliferation to antigen	Recurrent/chronic bacterial and viral infections (EBV, VZV), EBV lymphoproliferation, B cell non-Hodgkin lymphoma
CD137 deficiency (41BB)	*TNFRSF9*	AR	602250	Normal	Normal	Low IgG, low IgA, poor responses to T cell-dependent and T cell independent antigens, decreased T cell proliferation, IFNγ secretion, cytotoxicity	EBV lymphoproliferation, B cell lymphoma, chronic active EBV infection
RASGRP1 deficiency	*RASGRP1*	AR	603962	Poor activation, proliferation, motility. Reduced naïve T cells	Poor activation, proliferation, motility	Normal IgM, IgG, increased IgA	Recurrent pneumonia, herpesvirus infections, EBV associated lymphoma Decreased NK cell function
RLTPR deficiency	*CARMIL2*	AR	610859	Normal number, high CD4, increased naïve CD4^+^ and CD8^+^ T cells, low Treg and MAIT, poor CD28-induced function	Normal B cell numbers, reduced memory B cells	Normal to low, poor T dependent antibody response	Recurrent bacterial, fungal and mycobacterial infections, viral warts, molluscum and EBV lymphoproliferative and other malignancy, atopy
X-linked magnesium EBV and neoplasia (XMEN)	*MAGT1*	XL	300853	Low CD4 Low recent thymic emigrant cels, inverted CD4/CD8 ratio, reduced MAIT cells, poor proliferation to CD3	Normal but decreased memory B cells	Progressive hypogammaglobulinemia Reduced NK cell and CTL cytotoxic activity due to impaired expression of NKG2D	EBV infection, lymphoma, viral infections, respiratory and GI infections Glycosylation defects
PRKCD deficiency	*PRKCD*	AR	615559	Normal	Low memory B cells, high CD5 B cells	Apoptotic defect in B cells	Recurrent infections, EBV chronic infection, lymphoproliferation, SLE-like autoimmunity (nephrotic and antiphospholipid syndromes), low IgG

Total number of disorders in Table 4: 44

Total number of mutant genes in Table 4: 45

New disorders: 8; *SLC7A7* [57]; *IL2RB* [58, 59]; *DEF6* [60]; *FERMT1* [61]; *TGFB1* [62]; *RIPK1* [63, 64]; *TNFRSF9* [46, 65, 66]; *STAT5B* AD DN []

*FHL* familial hemophagocytic lymphohistiocytosis, *HLH* hemophagocytic lymphohistiocytosis, *HSM* hepatosplenomegaly, *DN* double-negative, *SLE* systemic lupus erythematous, *IBD* Inflammatory bowel disease

**Table 5:** Congenital defects of phagocyte number or function

Disease	Genetic defect	Inheritance	OMIM	Affected cells	Affected function	Associated features
1. Congenital neutropenias						
Elastase deficiency (Severe congential neutropenia [SCN] 1)	*ELANE*	AD	130130	N	Myeloid differentiation	Susceptibility to MDS/leukemia Severe congenital neutropenia or cyclic neutropenia
GFI 1 deficiency (SCN2)	*GFI1*	AD	600871	N	Myeloid differentiation	B/T lymphopenia
HAX1 deficiency (Kostmann Disease) (SCN3)	*HAX1*	AR	605998	N	Myeloid differentiation	Cognitive and neurological defects in patients with defects in both HAX1 isoforms, susceptibility to MDS/leukemia
G6PC3 deficiency (SCN4)	*G6PC3*	AR	611045	N	Myeloid differentiation, chemotaxis, O_2_^−^ production	Structural heart defects, urogenital abnormalities, inner ear deafness, and venous angiectasias of trunks and limbs
VPS45 deficiency (SCN5)	*VPS45*	AR	610035	N	Myeloid differentiation, migration	Extramedullary hematopoiesis, bone marrow fibrosis, nephromegaly
Glycogen storage disease type 1b	*G6PT1*	AR	602671	N + M	Myeloid differentiation, chemotaxis, O_2_^−^ production	Fasting hypoglycemia, lactic acidosis, hyperlipidemia, hepatomegaly
X-linked neutropenia/myelodysplasia	*WAS*	XL GOF	300299	N	Differentiation, mitosis. Results from GOF mutations in GTPase binding domain of WASp	Neutropenia, myeloid maturation arrest, monocytopenia, variable lymphoid anomalies
P14/LAMTOR2 deficiency	*LAMTOR2*	AR	610389	N + M	Endosomal biogenesis	Neutropenia Hypogammaglobulinemia ↓CD8 cytotoxicity, partial albinism, growth failure
Barth Syndrome (3-Methylglutaconic aciduria type II)	*TAZ*	XL	300394	N + L Mel	Mitochondrial function	Cardiomyopathy, myopathy, growth retardation, neutropenia
Cohen syndrome	*VPS13B*	AR	607817	N	Myeloid differentiation	Dysmorphism, mental retardation, obesity, deafness, neutropenia
Clericuzio syndrome (Poikiloderma with neutropenia)	*USB1*	AR	613276	N	Myeloid differentiation	Retinopathy, developmental delay, facial dysmorphisms, poikiloderma
JAGN1 deficiency	*JAGN1*	AR	616012	N	Myeloid differentiation	Myeloid maturation arrest, osteopenia
3-Methylglutaconic aciduria	*CLPB*	AR	616254	N	Myeloid differentiation Mitochondrial protein	Neurocognitive developmental aberrations, microcephaly, hypoglycemia, hypotonia, ataxia, seizures, cataracts, IUGR
G-CSF receptor deficiency	*CSF3R*	AR	138971	N	Stress granulopoiesis disturbed	
SMARCD2 deficiency	*SMARCD2*	AR	601736	N	Chromatin remodeling, Myeloid differentiation and neutrophil functional defect	Neutropenia, developmental aberrations, bones, hematopoietic stem cells, myelodysplasia
Specific granule deficiency	*CEBPE*	AR	189965	N	Terminal maturation and global dysfunction	Neutropenia, Neutrophils with bilobed nuclei
Shwachman-Diamond Syndrome	*SBDS*	AR	607444	N	Neutrophil maturation, chemotaxis, ribosomal biogenesis	Pancytopenia, exocrine pancreatic insufficiency, chondrodysplasia
	*DNAJC21*	AR	617052	N + HSC		Pancytopenia, exocrine pancreatic insufficiency
	*EFL1*	AR	617941	N + HSC		
HYOU1 deficiency	*HYOU1*	AR	601746	N	Unfolded protein response	Hypoglycemia, inflammatory complications
SRP54 deficiency	*SRP54*	AD	604857	N	Protein translocation to ER, myeloid differentiation and neutrophil functional defect	Neutropenia, exocrine pancreatic insufficiency
2. Defects of motility						
Leukocyte adhesion deficiency type 1 (LAD1)	*ITGB2*	AR	600065	N + M + L + NK	Adherence, chemotaxis, endocytosis, T/NK cytotoxicity	Delayed cord separation, skin ulcers, periodontitis, leukocytosis
Leukocyte adhesion deficiency type 2 (LAD2)	*SLC35C1*	AR	605881	N + M	Rolling, chemotaxis	Mild LAD type 1 features with hh-blood group, growth retardation, developmental delay
Leukocyte adhesion deficiency type 3 (LAD3)	*FERMT3*	AR	607901	N + M + L + NK	Adherence, chemotaxis	LAD type 1 plus bleeding tendency
Rac2 deficiency	*RAC2*	AD LOF	608203	N	Adherence, chemotaxis O_2_− production	Poor wound healing, leukocytosis
β actin deficiency	*ACTB*	AD	102630	N + M	Motility	Mental retardation, short stature
Localized juvenile periodontitis	*FPR1*	AR	136537	N	Formylpeptide induced chemotaxis	Periodontitis only
Papillon-Lefèvre syndrome	*CTSC*	AR	602365	N + M	Chemotaxis	Periodontitis, palmoplantar hyperkeratosis in some patients
WDR1 deficiency	*WDR1*	AR	604734	N	Spreading, survival, chemotaxis	Mild neutropenia, poor wound healing, severe stomatitis, neutrophil nuclei herniate
Cystic fibrosis	*CFTR*	AR	602421	M only	Chemotaxis	Respiratory infections, pancreatic insufficiency, elevated sweat chloride
Neutropenia with combined immune deficiency due to MKL1 deficiency	*MKL1*	AR	606078	N + M + L + NK	Impaired expression of cytoskeletal genes	Mild thrombocytopenia
3. Defects of respiratory burst						
X-linked chronic granulomatous disease (CGD), gp91phox	*CYBB*	XL	306400	N + M	Killing (faulty O_2_− production)	Infections, autoinflammatory phenotype, IBD McLeod phenotype in patients with deletions extending into the contiguous Kell locus
Autosomal recessive CGD	*CYBA*	AR	608508			Infections, autoinflammatory phenotype
	*CYBC1*		618334			
	*NCF1*		608512			
	*NCF2*		608515			
	*NCF4*		613960			
G6PD deficiency class I	*G6PD*	XL	305900	N	Reduced O2− production	Infections
4. Other non-lymphoid defects						
GATA2 deficiency	*GATA2*	AD	137295	Monocytes + peripheral DC	Multi lineage cytopenias	Susceptibility to mycobacteria, HPV, histoplasmosis, alveolar proteinosis, MDS/AML/CMML, lymphedema
Pulmonary alveolar proteinosis	*CSF2RA*	XL (Biallelic mutations in pseudo-autosomal gene)	300770	Alveolar macrophages	GM-CSF signaling	Alveolar proteinosis
	*CSFR2B*	AR	614370			

Total number of disorders in Table 5: 34

Total number of mutant genes in Table 5: 41

New disorders: 3; *SRP54* [67, 68]; *DNAJC21* [69]; *CYBC1* [70, 71]

Removed: Cyclic neutropenia was merged with elastase deficiency

*MDS* myelodysplastic syndrome, *IUGR* intrauterine growth retardation, *LAD* leukocyte adhesion deficiency, *AML* acute myelogenous leukemia, *CMML* chronic myelomonocytic leukemia, *N* neutrophil, *M* monocyte, *MEL* melanocyte, *L* lymphocyte, *NK* natural killer

**Table 6:** Defects in intrinsic and innate immunity

Disease	Genetic defect	Inheritance	OMIM	Affected cells	Affected function	Associated features
1. Mendelian susceptibility to mycobacterial disease (MSMD)						
IL-12 and IL-23 receptor β1 chain deficiency	*IL12RB1*	AR	601604	L + NK	IFN-γ secretion	Susceptibility to mycobacteria and *Salmonella*
IL-12p40 (IL-12 and IL-23) deficiency	*IL12B*	AR	161561	M		
IL-12Rβ2 deficiency	*IL12RB2*	AR	601642	L + NK		
IL-23R deficiency	*IL23R*	AR	607562	L + NK		
IFN-γ receptor 1 deficiency	*IFNGR1*	AR	209950	M + L	IFN-γ binding and signaling	
		AD	615978	M + L		
IFN-γ receptor 2 deficiency	*IFNGR2*	AR	147569	M + L	IFN-γ signaling	
STAT1 deficiency	*STAT1*	AD LOF	614892	M + L		
Macrophage gp91 phox deficiency	*CYBB*	XL	300645	Macrophage only	Killing (faulty O_2_− production)	Isolated susceptibility to mycobacteria
IRF8 deficiency	*IRF8*	AD	614893	M + L	Impaired development of cDCs and Th1* cells	Susceptibility to mycobacteria
		AR	226990	M	Lack of circulating monocytes and DCs, reduced NK cell numbers and function reported in some patients	Susceptibility to mycobacteria and multiple other infectious agents including EBV
SPPL2a deficiency	*SPPL2A*	AR	608238	M + L	Impaired development of cDCs and Th1* cells	Susceptibility to mycobacteria and *Salmonella*
Tyk2 deficiency	*TYK2*	AR	611521	M + L	Impaired cellular responses to IL-10, IL-12, IL-23, and type I IFNs	Susceptibility to intracellular bacteria (mycobacteria, Salmonella), and viruses
P1104A TYK2 homozygosity	*TYK2*	AR	176941	L	Impaired cellular responses to IL-23	MSMD or tuberculosis
ISG15 deficiency	*ISG15*	AR	147571		IFNγ production defect	Susceptibility to mycobacteria (BCG), brain calcification
RORγt deficiency	*RORC*	AR	602943	L + NK	Lack of functional RORγT protein, IFNγ production defect, complete absence of IL-17A/F-producing T cells	Susceptibility to mycobacteria and candida
JAK1 deficiency	*JAK1*	AR LOF	147795	N + L	Reduced JAK1 activation to cytokines, Reduced IFNγ production	Susceptibility to mycobacteria and viruses, urothelial carcinoma
2. Epidermodysplasia verruciformis (HPV)						
EVER1 deficiency	*TMC6*	AR	605828	Keratinocytes	EVER1, EVER2 and CIB1 form a complex in keratinocytes	Human papillomavirus (HPV) (group B1) infections and cancer of the skin (typical EV)
EVER2 deficiency	*TMC8*		605829			
CIB1 deficiency	*CIB1*		618267			
WHIM (warts, hypogammaglobulinemia, infections, myelokathexis) syndrome	*CXCR4*	AD GOF	162643	Leukocytes	Increased response of the CXCR4 chemokine receptor to its ligand CXCL12 (SDF-1)	Warts (HPV) infection, neutropenia, low B cell number, hypogammaglobulinemia
3. Predisposition to severe viral infection						
STAT1 deficiency	*STAT1*	AR LOF	600555	Leukocytes and other cells	STAT1-dependent IFN-α/β, γ and λ responses	Severe viral infections, mycobacterial infection
STAT2 deficiency	*STAT2*	AR	600556	Leukocytes and other cells	STAT2-dependent IFN-α/β and λ response	Severe viral infections (disseminated vaccine-strain measles)
IRF9 deficiency	*IRF9*	AR	147574*	Leukocytes and other cells	IRF9- and ISGF3-dependent IFN-α/β and λ responses	Severe influenza disease
IRF7 deficiency	*IRF7*	AR	605047	Leukocytes, plasmacytoid dendritic cells, non-hematopoietic cells	IFN-α, β and γ production and IFN-λ production	
IFNAR1 deficiency	*IFNAR1*	AR	107450*	Leukocytes and other cells	IFNAR1-dependent responses to IFN-α/β	Severe disease caused by Yellow Fever vaccine and Measles vaccine
IFNAR2 deficiency	*IFNAR2*	AR	602376	Broadly expressed	IFNAR2-dependent responses to IFN-α/β	Severe viral infections (disseminated vaccine-strain measles, HHV6)
CD16 deficiency	*FCGR3A*	AR	146740	NK cells	Altered NK cells function	Severe herpes viral infections, particularly VZV, Epstein-Barr virus (EBV), and (HPV)
MDA5 deficiency	*IFIH1*	AR LOF	606951	Broadly expressed	Viral recognition and IFN induction	Rhinovirus and other RNA viruses
RNA polymerase III deficiency	*POLR3A*	AD	614258	Leukocytes and other cells	Impaired viral recognition and IFN induction in response to VZV or poly I:C	Severe VZV infection
	*POLR3C*	AD	617454			
	*POLR3F*	AD	617455			
4. Herpes simplex encephalitis (HSE)						
TLR3 deficiency	*TLR3*	AD	613002	Central nervous system (CNS) resident cells and fibroblasts	TLR3-dependent IFN-α, β and γ response	Herpes simplex virus 1 encephalitis (incomplete clinical penetrance for all etiologies listed here); severe pulmonary influenza; VZV
		AR				
UNC93B1 deficiency	*UNC93B1*	AR	608204		UNC-93B-dependent IFN-α, β and γ response	Herpes simplex virus 1 encephalitis
TRAF3 deficiency	*TRAF3*	AD	601896		TRAF3-dependent IFN-α, β and γ response	
TRIF deficiency	*TICAM1*	AD	607601		TRIF-dependent IFN-α, β and γ response	
		AR				
TBK1 deficiency	*TBK1*	AD	604834		TBK1-dependent IFN-α, β and γ response	
IRF3 deficiency	*IRF3*	AD	616532		Low IFN-α/β production in response to HSV1 and decreased IRF3 phosphorylation	
DBR1 deficiency	*DBR1*	AR	607024		Impaired production of anti-viral IFNs	HSE of the brainstem. Other viral infections of the brainstem.
5. Predisposition to invasive fungal diseases						
CARD9 deficiency	*CARD9*	AR	607212	Mononuclear phagocytes	CARD9 signaling pathway	Invasive candidiasis infection, deep dermatophytoses, other invasive fungal infections
6. Predisposition to mucocutaneous candidiasis						
IL-17RA deficiency	*IL17RA*	AR	605461	Epithelial cells, fibroblasts, mononuclear phagocytes	IL-17RA signaling pathway	CMC, folliculitis
IL-17RC deficiency	*IL17RC*	AR	610925		IL-17RC signaling pathway	CMC
IL-17F deficiency	*IL17F*	AD	606496	T cells	IL-17F-containing dimers	CMC, folliculitis
STAT1 GOF	*STAT1*	AD GOF	600555	T cells, B cells, monocytes	Gain-of-function STAT1 mutations that impair the development of IL-17-producing T cells	CMC, various fungal, bacterial and viral (HSV) infections, auto-immunity (thyroiditis, diabetes, cytopenias), enteropathy
ACT1 deficiency	*TRAF3IP2*	AR	607043	T cells, fibroblasts	Fibroblasts fail to respond to IL-17A and IL-17F, and their T cells to IL-17E	CMC, blepharitis, folliculitis, and macroglossia
7. TLR signaling pathway deficiency with bacterial susceptibility						
IRAK4 deficiency	*IRAK4*	AR	606883	Lymphocytes + granulocytes+ monocytes	TIR-IRAK4 signaling pathway	Bacterial infections (pyogens)
MyD88 deficiency	*MYD88*	AR	602170	Lymphocytes + granulocytes + monocytes	TIR-MyD88 signaling pathway	
IRAK1 deficiency	*IRAK1*	XL	300283	Lymphocytes + granulocytes + monocytes	TIR-IRAK1 signaling pathway	Bacterial infections, X-linked MECP2 deficiency-related syndrome due to a large de novo Xq28 chromosomal deletion encompassing both *MECP2* and *IRAK1*
TIRAP deficiency	*TIRAP*	AR	614382	Lymphocytes + granulocytes + monocytes	TIRAP- signaling pathway, TLR1/2, TLR2/6, and TLR4 agonists were impaired in the fibroblasts and leukocytes	Staphylococcal disease during childhood
8. Other inborn errors of immunity related to non-hematopoietic tissues						
Isolated congenital asplenia (ICA)	*RPSA*	AD	271400	No spleen	RPSA encodes ribosomal protein SA, a component of the small subunit of the ribosome	Bacteremia (encapsulated bacteria)
	*HMOX*	AR	141250	Macrophages	HO-1 regulates iron recycling and heme-dependent damage occurs	Hemolysis, nephritis, inflammation
Trypanosomiasis	*APOL1*	AD	603743	Somatic	Pore forming serum protein	Trypanosomiasis
Acute liver failure due to NBAS deficiency	*NBAS*	AR	608025	Somatic and hematopoietic	ER stress	Fever induces liver failure
Acute necrotizing encephalopathy Osteopetrosis	*RANBP2*	AR	601181	Ubiquitous expression	Nuclear pore	Fever induces acute encephalopathy
	*CLCN7*	AR	602727	Osteoclasts	Secretory lysosomes	Osteopetrosis with hypocalcemia, neurologic features
	*SNX10*	AR	614780			Osteopetrosis with visual impairment
	*OSTM1*	AR	607649			Osteopetrosis with hypocalcemia, neurologic features
	*PLEKHM1*	AR	611466			Osteopetrosis
	*TCIRG1*	AR	604592			Osteopetrosis with hypocalcemia
	*TNFRSF11A*	AR	603499		Osteoclastogenesis	Osteopetrosis
	*TNFSF11*	AR	602642	Stromal	Osteoclastogenesis	Osteopetrosis with severe growth retardation
Hidradenitis suppurativa	*NCSTN*	AD	605254	Epidermis	Notch signaling/gamma-secretase in hair follicle regulates keratinization	Verneuil’s disease/Hidradenitis suppurativa with acne
	*PSEN*	AD	613737			Verneuil’s disease/Hidradenitis suppurative with cutaneous hyperpigmentation
	*PSENEN*	AD	613736			Verneuil’s disease/Hidradenitis suppurativa
9. Other inborn errors of immunity related to leukocytes						
IRF4 haploinsufficiency	*IRF4*	AD	601900	L + M	IRF4 is a pleiotropic transcription factor	Whipple’s disease
IL-18BP deficiency	*IL18BP*	AR	604113	Leukocytes and other cells	IL-18BP neutralizes secreted IL-18	Fulminant viral hepatitis

Total number of disorders in Table 6: 53

Total number of mutant genes in Table 6: 64

New genes: 13, *IL12RB2* [72]; *IL23R* [72]; *SPPL2A* [73]; *TYK2 P1104A allele* [10]; *CIB1* [74]; *IRF9* [75]; *IFNAR1* [76]; *POLR3A* [77]; *POLR3C* [77]; *POLR3F* [78]; *DBR1* [79]; *IRF4* [80]; *IL18BP* [81]

*NF-κB* nuclear factor kappa B, *TIR* Toll and Interleukin 1 receptor, *IFN* interferon, *TLR* Toll-like receptor, *MDC* myeloid dendritic cell, *CNS* central nervous system, *CMC* chronic mucocutaneous candidiasis, *HPV* human papillomavirus, *VZV* varicella zoster virus, *EBV*, Epstein-Barr virus

**Table 7:** Autoinflammatory disorders

Disease	Genetic defect	Inheritance	OMIM	T cells	B cells	Functional defect	Associated features
1. Type 1 interferonopathies							
STING-associated vasculopathy, infantile-onset (SAVI)	*TMEM173*	AR	612374	Not assessed	Not assessed	STING activates both the NF-kappa-B and IRF3 transcription pathways to induce expression of IFN	Skin vasculopathy, inflammatory lung disease, systemic autoinflammation and ICC, FCL
ADA2 deficiency	*ADA2*	AR	607575	Not assessed	Not assessed	ADAs deactivate extracellular adenosine and terminate signaling through adenosine receptors	Polyarteritis nodosa, childhood-onset, early-onset recurrent ischemic stroke and fever; some patients develop hypogammaglobulinemia
TREX1 deficiency, Aicardi-Goutieres syndrome 1 (AGS1)	*TREX1*	AR	606609	Not assessed	Not assessed	Intracellular accumulation of abnormal ss DNA species leading to increased type I IFN production	Classical AGS, SLE, FCL
RNASEH2B deficiency, AGS2	*RNASEH2B*	AR	610326	Not assessed	Not assessed	Intracellular accumulation of abnormal RNA-DNA hybrid species leading to increased type I IFN production	Classical AGS, SP
RNASEH2C deficiency, AGS3	*RNASEH2C*	AR	610330	Not assessed	Not assessed		Classical AGS
RNASEH2A deficiency, AGS4	*RNASEH2A*	AR	606034	Not assessed	Not assessed		Classical AGS
SAMHD1 deficiency, AGS5	*SAMHD1*	AR	606754	Not assessed	Not assessed	Controls dNTPs in the cytosol, failure of which leads to increased type I IFN production	Classical AGS, FCL
ADAR1 deficiency, AGS6	*ADAR1*	AR	146920	Not assessed	Not assessed	Catalyzes the deamination of adenosine to inosine in dsRNA substrates, failure of which leads to increased type I IFN production	Classical AGS, BSN, SP
Aicardi-Goutieres syndrome 7 (AGS7)	*IFIH1*	AD GOF	615846	Not assessed	Not assessed	IFIH1 gene encodes a cytoplasmic viral RNA receptor that activates type I interferon signaling through the MAVS adaptor molecule	Classical AGS, SLE, SP, SMS
DNAse II deficiency	*DNASE2*	AR	126350	Not assessed	Not assessed	DNAse II degrades and eliminates DNA. Loss of DNase II activity induces type I interferon signaling	AGS
Pediatric systemic lupus erythematosus due to DNASE1L3 deficiency	*DNASE1L3*	AR	614420			DNASE1L3 is an endonuclease that degrades extracellular DNA. DNASE1L3 deficiency decreases clearance of apoptotic cells	Very early onset SLE, reduced complement levels, autoantibodies (dsDNA, ANCA), lupus nephritis, hypocomplementemic urticarial vasculitis syndrome
Spondyloenchondro-dysplasia with immune dysregulation (SPENCD)	*ACP5*	AR	171640	Not assessed	Not assessed	Upregulation of IFN through mechanism possibly relating to pDCS	Short stature, SP, ICC, SLE, thrombocytopenia and autoimmune hemolytic anemia, possibly recurrent bacterial and viral infections
X-linked reticulate pigmentary disorder	*POLA1*	XL	301220	Not assessed	Not assessed	POLA1 is required for synthesis of cytosolic RNA:DNA and its deficiency leads to increase production of type I interferon	Hyperpigmentation, characteristic facies, lung and GI involvement
USP18 deficiency	*USP18*	AR	607057	Not assessed	Not assessed	Defective negative regulation of ISG15 leading to increased IFN	TORCH-like syndrome
OAS1 deficiency	*OAS1*	AD GOF	164350		Low	Increased interferon from recognition of RNA	Pulmonary alveolar proteinosis, skin rash
2. Defects affecting the inflammasome							
Familial Mediterranean fever	*MEFV*	AR LOF	249100	Mature granulocytes, cytokine-activated monocytes.	Increased inflammasome-mediated induction of IL1β.	Recurrent fever, serositis and inflammation responsive to colchicine. Predisposes to vasculitis and inflammatory bowel disease.	
		AD	134610	Mature granulocytes, cytokine-activated monocytes.	Usually M694del variant.		
Mevalonate kinase deficiency (Hyper IgD syndrome)	*MVK*	AR	260920	Somatic and hemaotpoietic	affecting cholesterol synthesis, pathogenesis of disease unclear	Periodic fever and leukocytosis with high IgD levels	
Muckle-Wells syndrome	*NLRP3*	AD GOF	191900	PMNs Monocytes	Defect in cryopyrin, involved in leukocyte apoptosis and NFkB signaling and IL-1 processing	Urticaria, SNHL, amyloidosis.	
Familial cold autoinflammatory syndrome 1		AD GOF	120100	PMNs, monocytes		Non-pruritic urticaria, arthritis, chills, fever and leukocytosis after cold exposure.	
Neonatal onset multisystem inflammatory disease (NOMID) or chronic infantile neurologic cutaneous and articular syndrome (CINCA)		AD GOF	607115	PMNs, chondrocytes		Neonatal onset rash, chronic meningitis, and arthropathy with fever and inflammation.	
Familial cold autoinflammatory syndrome 2	*NLRP12*	AD GOF	611762	PMNs, monocytes		Non-pruritic urticaria, arthritis, chills, fever and leukocytosis after cold exposure.	
NLRC4-MAS (macrophage activating syndrome)	*NLRC4*	AD GOF	616050	PMNs monocytes macrophages	Gain of function mutation in *NLRC4* results in elevated secretion of IL-1β and IL-18 as well as macrophage activation	Severe enterocolitis and macrophage activation syndrome	
Familial cold autoinflammatory syndrome 4			616115				
PLAID (PLCγ2 associated antibody deficiency and immune dysregulation)	*PLCG2*	AD GOF	614878	B cells, NK, Mast cells	Mutations activate IL-1 pathways	Cold urticaria hypogammaglobulinemia, impaired humoral immunity, autoinflammation	
Familial cold autoinflammatory syndrome 3 or APLAID (c2120A > C)			614468				
NLRP1 deficiency	*NLRP1*	AR	617388	leukocytes	Systemic elevation of IL-18 and caspase 1, suggesting involvement of NLRP1 inflammasome	Dyskeratosis, autoimmunity and arthritis	
NLRP1 GOF	*NLRP1*	AD GOF	615225	Keratinocytes	Increased IL1β	Palmoplantar carcinoma, corneal scarring; recurrent respiratory papillomatosis	
3. Non-inflammasome-related conditions							
TNF receptor-associated periodic syndrome (TRAPS)	*TNFRSF1A*	AD	142680	PMNs, monocytes	Mutations of 55-kD TNF receptor leading to intracellular receptor retention or diminished soluble cytokine receptor available to bind TNF	Recurrent fever, serositis, rash, and ocular or joint inflammation	
Pyogenic sterile arthritis, pyoderma gangrenosum, acne (PAPA) syndrome, hyperzincemia and hypercalprotectinemia	*PSTPIP1*	AD	604416	Hematopoietic tissues, upregulated in activated T cells	Disordered actin reorganization leading to compromised physiologic signaling during inflammatory response	Destructive arthritis, inflammatory skin rash, myositis	
Blau syndrome	*NOD2*	AD	186580	Monocytes	Mutations in nucleotide binding site of CARD15, possibly disrupting interactions with lipopolysaccharides and NF-kB signaling	Uveitis, granulomatous synovitis, camptodactyly, rash and cranial neuropathies, 30% develop Crohn colitis	
ADAM17 deficiency	*ADAM17*	AR	614328	Leukocytes and epithelial cells	Defective TNFα production	Early onset diarrhea and skin lesions	
Chronic recurrent multifocal osteomyelitis and congenital dyserythropoietic anemia (Majeed syndrome)	*LPIN2*	AR	609628	Neutrophils, bone marrow cells	Undefined	Chronic recurrent multifocal osteomyelitis, transfusion-dependent anemia, cutaneous inflammatory disorders	
DIRA (Deficiency of the Interleukin 1 Receptor Antagonist)	*IL1RN*	AR	612852	PMNs, Monocytes	Mutations in the IL1 receptor antagonist allow unopposed action of Interleukin 1	Neonatal onset of sterile multifocal osteomyelitis, periostitis and pustulosis.	
DITRA (Deficiency of IL-36 receptor antagonist)	*IL36RN*	AR	614204	Keratinocytes, leukocytes	Mutations in IL-36RN leads to increase IL-8 production	Pustular psoriasis	
SLC29A3 mutation	*SLC29A3*	AR	602782	Leukocytes, bone cells	–	Hyperpigmentation hypertrichosis, histiocytosis-lymphadenopathy plus syndrome	
CAMPS (CARD14 mediated psoriasis)	*CARD14*	AD	602723	Mainly in keratinocytes	Mutations in CARD14 activate the NF-*k*B pathway and production of IL-8	Psoriasis	
Cherubism	*SH3BP2*	AD	118400	Stroma cells, bone cells	Hyperactived macrophage and increase NF-kB	Bone degeneration in jaws	
CANDLE (chronic atypical neutrophilic dermatitis with lipodystrophy)	PSMB8*	AR and AD	256040	Keratinocytes, B cell adipose cells	Mutations cause increased IFN signaling through an undefined mechanism	Contractures, panniculitis, ICC, fevers	
	*PSMG2*	AR	609702	Lymphocytes		Panniculitis, lipodystrophy, autoimmune hemolytic anemia	
COPA defect	*COPA*	AD	6011924	PMN and tissue specific cells	Defective intracellular transport via the coat protein complex I (COPI)	Autoimmune inflammatory arthritis and interstitial lung disease with Th17 dysregulation and autoantibody production	
Otulipenia/ORAS	*OTULIN*	AR	615712	Leukocytes	Increase LUBAC induction of NF-KB activation leading to high proinflamatory cytokines levels.	Fever, diarrhea, dermatitis	
A20 deficiency	*TNFAIP3*	AD	616744	Lymphocytes	Defective inhibition of NF-KB signaling pathway	Arthralgia, mucosal ulcers, ocular inflammation	
AP1S3 deficiency	*AP1S3*	AR	615781	Keratinocytes	Disrupted TLR3 translocation	Pustular psoriasis	
ALPI deficiency	*ALPI*	AR	171740	Intestinal epithelial cells	Deficient inhibition of LPS in intestine	Inflammatory bowel disease	
TRIM22	*TRIM22*	AR	606559	Macrophages, intestinal epithelial cells	Granulomatous colitis	Inflammatory bowel disease	
T cell lymphoma subcutaneous panniculitis-like (TIM3 deficiency)	*HAVCR2*	AR	618398	Leukocytes	Increased inflammasome activity due to defective checkpoint signaling	Panniculitis, HLH, polyclonal cutaneous T cell infiltrates or T cell lymphoma	

Total number of disorders in Table 7: 45

Total number of mutant genes in Table 7: 42

New disorders: 9; *DNASE2* [82]; *DNASE1L3* [83–85]; *OAS1* [86]; AD *MEFV; NLRP1 GOF* [87, 88]; *ALPI* [89]; *TRIM22* [90]; *PSMG2* [91]; *HAVCR2* [92, 93]

*IFN* interferon, *HSM* hepatosplenomegaly, *CSF* cerebrospinal fluid, *SLE* systemic lupus erythematosus, *TORCH* toxoplasmosis, other, rubella, cytomegalovirus, and herpes infections, *SNHL* sensorineural hearing loss, *AGS* Aicardi-Goutières syndrome, *BSN* bilateral striatal necrosis, *FCL* familial chilblain lupus, *ICC* intracranial calcification, *IFN* interferon type I, *pDCs* plasmacytoid dendritic cells, *SP* spastic paraparesis, *SMS* Singleton-Merten syndrome, *ss* single-stranded DNA

*Variants in *PSMB4*, *PSMB9*, *PSMA3*, and *POMP* have been proposed to cause a similar CANDLE phenotype in compound heterozygous monogenic (*PSMB4*), digenic (*PSMA3/PSMB8*, *PSMB9/PSMB4*, *PSMB4/PSMB8*) and AD monogenic (*POMP*) models [94]

**Table 8:** Complement deficiencies

Disease	Genetic defect	Inheritance	Gene OMIM	Laboratory features	Associated features
Complement deficiencies					
C1q deficiency due to defects	*C1QA*	AR	120550	Absent CH50 hemolytic activity, defective activation of the classical pathway, diminished clearance of apoptotic cells	SLE, infections with encapsulated organisms
	*C1QB*	AR	120570		
	*C1QC*	AR	120575		
C1r deficiency	*C1R*	AR	613785	Absent CH50 hemolytic activity, defective activation of the classical pathway	SLE, infections with encapsulated organisms, Ehlers-Danlos phenotype
C1r Periodontal Ehlers-Danlos	*C1R*	AD GOF	613785	Normal CH50	Hyperpigmentation, skin fragility
C1s deficiency	*C1S*	AR	613785	Absent CH50 hemolytic activity, defective activation of the classical pathway	SLE, infections with encapsulated organisms, Ehlers-Danlos phenotype
C1s Periodontal Ehlers-Danlos	*C1S*	AD GOF	613785	Normal CH50	Hyperpigmentation, skin fragility
Complete C4 deficiency	*C4A + C4B*	AR	120810	Absent CH50 hemolytic activity, defective activation of the classical pathway, complete deficiency requires biallelic mutations/deletions/conversions of both C4A and C4B	SLE, infections with encapsulated organisms, partial deficiency is common (either C4A or C4B) and appears to have a modest effect on host defense
C2 deficiency	*C2*	AR	217000	Absent CH50 hemolytic activity, defective activation of the classical pathway	SLE, infections with encapsulated organisms, atherosclerosis
C3 deficiency (LOF)	*C3*	AR	120700	Absent CH50 and AH50 hemolytic activity, defective opsonization, defective humoral immune response	Infections, glomerulonephritis, atypical hemolytic-uremic syndrome with GOF mutations.
C3 GOF	*C3*	AD GOF	120700	Increased activation of complement	Atypical hemolytic-uremic syndrome
C5 deficiency	*C5*	AR	120900	Absent CH50 and AH50 hemolytic activity Defective bactericidal activity	Disseminated neisserial infections
C6 deficiency	*C6*	AR	217050	Absent CH50 and AH50 hemolytic activity, defective bactericidal activity	
C7 deficiency	*C7*	AR	217070		
C8α deficiency	*C8A*	AR	120950		
C8 γ deficiency	*C8G*	AR	120930		
C8 β deficiency	*C8B*	AR	120960		
C9 deficiency	*C9*	AR	120940	Reduced CH50 and AP50 hemolytic activity, deficient bactericidal activity	Mild susceptibility to disseminated neisserial infections
MASP2 deficiency	*MASP2*	AR	605102	Deficient activation of the lectin activation pathway	Pyogenic infections, inflammatory lung disease, autoimmunity
Ficolin 3 deficiency	*FCN3*	AR	604973	Absence of complement activation by the Ficolin 3 pathway.	Respiratory infections, abscesses
C1 inhibitor deficiency	*SERPING1*	AD	606860	Spontaneous activation of the complement pathway with consumption of C4/C2, spontaneous activation of the contact system with generation of bradykinin from high molecular weight kininogen	Hereditary angioedema
Factor B GOF	*CFB*	AD GOF	612924	Gain-of-function mutation with increased spontaneous AH50	Atypical hemolytic-uremic syndrome
Factor B deficiency	*CFB*	AR	615561	Deficient activation of the alternative pathway	Infections with encapsulated organisms
Factor D deficiency	*CFD*	AR	134350	Absent AH50 hemolytic activity	Neisserial infections
Properdin deficiency	*CFP*	XL	300383	Absent AH50 hemolytic activity	Neisserial infections
Factor I deficiency	*CFI*	AR	217030	Spontaneous activation of the alternative complement pathway with consumption of C3	Infections, disseminated neisserial infections, atypical Hemolytic-uremic syndrome, preeclampsia
Factor H deficiency	*CFH*	AR or AD	134370	Spontaneous activation of the alternative complement pathway with consumption of C3	
Factor H-related protein deficiencies	*CFHR1*	AR or AD	134371,	Normal CH50, AH50, autoantibodies to Factor H., linked deletions of one or more CFHR genes leads to susceptibility autoantibody-mediated aHUS	Older onset atypical hemolytic-uremic syndrome, disseminated neisserial infections
	*CFHR2*		600889,		
	*CFHR3*		605336,		
	*CFHR4*		605337,		
	*CFHR5*		608593		
Thrombomodulin deficiency	*THBD*	AD	188040	Normal CH50, AH50	Atypical hemolytic-uremic syndrome
Membrane Cofactor Protein (CD46) deficiency	*CD46*	AD	120920	Inhibitor of complement alternate pathway, decreased C3b binding	Atypical hemolytic-uremic syndrome, infections, preeclampsia
Membrane Attack Complex Inhibitor (CD59) deficiency	*CD59*	AR	107271	Erythrocytes highly susceptible to complement-mediated lysis	Hemolytic anemia, polyneuropathy
CD55 deficiency (CHAPEL disease)	*CD55*	AR	125240	Hyperactivation of complement on endothelium	Protein losing enteropathy, thrombosis

Total number of disorders in Table 8: 30

Total number of mutant genes in Table 8: 36

New disorders: 2; *C1S* AD GOF [95], *C1R* AD GOF [95]

*MAC* membrane attack complex, *SLE* systemic lupus erythematosus

**Table 9:** Bone marrow failure

Disease	Genetic defect	Inheritance	Gene OMIM	T cells	B cells	Other affected cells	Associated features	**Major Category**	**Subcategory**
Bone marrow failure									
Fanconi anemia type A	*FANCA*	AR	227650	Normal to low	Normal to low	HSC	Normal to low NK, CNS, skeletal, skin, cardiac, GI, urogenital anomalies, increased chromosomal breakage	Bone marrow failure with immune deficiency	Fanconi Anemia
Fanconi anemia type B	*FANCB*	XLR	300514						
Fanconi anemia type C	*FANCC*	AR	227645						
Fanconi anemia type D1	*BRCA2*	AR	605724						
Fanconi anemia type D2	*FANCD2*	AR	227646						
Fanconi anemia type E	*FANCE*	AR	600901						
Fanconi anemia type F	*FANCF*	AR	603467						
Fanconi anemia type G	*XRCC9*	AR	614082						
Fanconi anemia type I	*FANCI*	AR	609053						
Fanconi anemia type J	*BRIP1*	AR	609054						
Fanconi anemia type L	*FANCL*	AR	614083						
Fanconi anemia type M	*FANCM*	AR	618096						
Fanconi anemia type N	*PALB2*	AR	610832						
Fanconi anemia type O	*RAD51C*	AR	613390						
Fanconi anemia type P	*SLX4*	AR	613951						
Fanconi anemia type Q	*ERCC4*	AR	615272						
Fanconi anemia type R	*RAD51*	AR	617244						
Fanconi anemia type S	*BRCA1*	AR	617883						
Fanconi anemia type T	*UBE2T*	AR	616435						
Fanconi anemia type U	*XRCC2*	AR	617247						
Fanconi anemia type V	*MAD2L2*	AR	617243						
Fanconi anemia type W	*RFWD3*	AR	617784						
MIRAGE (myelodysplasia, infection, restriction of growth, adrenal hypoplasia, genital phenotypes, enteropathy)	*SAMD9*	AD GOF	617053	Not reported	Not reported	HSC, myeloid cells	Intrauterine growth retardation, gonadal abnormalities, adrenal failure, MDS with chromosome 7 aberrations, predisposition to infections, enteropathy, absent spleen		
Ataxia pancytopenia syndrome	*SAMD9L*	AD GOF	611170	Normal	Low	HSC, myeloid cells	MDS, neurological features		
DKCX1	*DKC1*	XL	305000	Normal to low	Normal to low	HSC	Bone marrow failure, pulmonary and hepatic fibrosis, nail dystrophy, leukoplakia, reticulate skin pigmentation; microcephaly, neurodevelopmental delay		Dyskeratosis Congenita
DKCA1	*TERC*	AD	127550						
DKCA2	*TERT*	AD	187270						
DKCA3	*TINF2*	AD	604319						
DKCA4	*RTEL1*	AD	616373						
DKCA5	*TINF2*	AD	268130						
DKCA6	*ACD*	AD	616553						
DKCB1	*NOLA3*	AR	224230						
DKCB2	*NOLA2*	AR	613987						
DKCB3	*WRAP53*	AR	613988						
DKCB4	*TERT*	AR	613989						
DKCB5	*RTEL1*	AR	615190		Low		Nail dystrophy, leukoplakia, bone marrow failure, severe B cell immunodeficiency, intrauterine growth retardation, growth retardation, microcephaly, cerebellar hypoplasia, and esophageal dysfunction		
DKCB6	*PARN*	AR	616353		Normal to low		Developmental delay, microcephaly, and cerebellar hypoplasia		
DKCB7	*ACD*	AR	616553		Normal to low		Bone marrow failure, pulmonary and hepatic fibrosis, nail dystrophy, leukoplakia, reticulate skin pigmentation; microcephaly, neurodevelopmental delay		
BMFS1 (SRP72-deficiency)	*SRP72*	AD	602122	NA	NA		Bone marrow failure and congenital nerve deafness		
BMFS2	*ERCC6L2*	AR	615667	NA	NA		Bone marrow failure, learning difficulties, microcephaly		
BMFS5	*TP53*	AD	618165	NA	Low B		Erythroid hypoplasia, B cell deficiency		
Coats plus syndrome	*STN1*	AR	613129	Normal	Normal		Intrauterine growth retardation, premature aging, pancytopenia, hypocellular bone marrow, gastrointestinal hemorrhage due to vascular ectasia, intracranial calcification, abnormal telomeres		
	*CTC1*	AR	617053	Not reported	Not reported				

Total number of disorders in Table 9: 43

Total number of mutant genes in Table 9: 43

*HSC* hematopoietic stem cell, *NK* natural killer, *CNS* central nervous system, *GI* gastrointestinal, *MDS* myelodysplastic syndrome, *DKCX* X-inked dyskeratosis congenital, *DKCA* autosomal dominant dyskeratosis congenita, *DKCB* autosomal recessive dyskeratosis congenita, *BMFS* bone marrow failure syndrome

**Table 10:** Phenocopies of inborn errors of immunity

Disease	Genetic defect/presumed pathogenesis	Circulating T cells	Circulating B cells	Serum Ig	Associated features/similar PID
1. Phenocopies of inborn errors of immunity					
Associated with somatic mutations					
Autoimmune lymphoproliferative syndrome (ALPS–SFAS)	Somatic mutation in *TNFRSF6*	Increased CD4^−^CD8^−^double negative (DN) αβ T cells	Normal, but increased number of CD5+ B cells	Normal or increased	Splenomegaly, lymphadenopathy, autoimmune cytopenias, Defective lymphocyte apoptosis/ALPS–FAS (=ALPS type Im)
RAS-associated autoimmune leukoproliferative disease (RALD)	Somatic mutation in *KRAS* (GOF)	Normal	B cell lymphocytosis	Normal or increased	Splenomegaly, lymphadenopathy, autoimmune cytopenias, granulocytosis, monocytosis/ALPS-like
RAS-associated autoimmune leukoproliferative disease (RALD)	Somatic mutation in *NRAS* (GOF)	Increased CD4−CD8− double negative (DN) T alpha/beta cells	Lymphocytosis	Normal or increased	Splenomegaly, lymphadenopathy, autoantibodies/ALPS-like
Cryopyrinopathy, (Muckle-Wells/CINCA/NOMID-like syndrome)	Somatic mutation in *NLRP3*	Normal	Normal	Normal	Urticaria-like rash, arthropathy, neurological signs
Hypereosinophilic syndrome due to somatic mutations in STAT5b	Somatic mutation in *STAT5B* (GOF)	Normal	Normal	Normal	Eosinophilia, atopic dermatitis, urticarial rash, diarrhea
Associated with autoantibodies					
Chronic mucocutaneous candidiasis	AutoAb to IL-17 and/or IL-22	Normal	Normal	Normal	Endocrinopathy, chronic mucocutaneous candidiasis/CMC
Adult-onset immunodeficiency with susceptibility to mycobacteria	AutoAb to IFNγ	Decreased naive T cells	Normal	Normal	Mycobacterial, fungal, *Salmonella* VZV infections/MSMD, or CID
Recurrent skin infection	AutoAb to IL-6	Normal	Normal	Normal	Staphylococcal infections/STAT3 deficiency
Pulmonary alveolar proteinosis	AutoAb to GM-CSF	Normal	Normal	Normal	Pulmonary alveolar proteinosis, cryptococcal meningitis, disseminated nocardiosis/CSF2RA deficiency
Acquired angioedema	AutoAb to CI inhibitor	Normal	Normal	Normal	Angioedema/*C1 INH* deficiency (hereditary angioedema)
Atypical hemolytic uremic syndrome	AutoAb to Complement Factor H	Normal	Normal	Normal	aHUS = Spontaneous activation of the alternative complement pathway
Thymoma with hypogammaglobulinemia (Good syndrome)	AutoAb to various cytokines	Increased CD8+ T cells	No B cells	Decreased	Invasive bacterial, viral or opportunistic infections, autoimmunity, PRCA, lichen planus, cytopenia, colitis, chronic diarrhea

*aHUS* atypical hemolytic uremic syndrome, *XL* X-linked inheritance, *AR* autosomal recessive inheritance, *AD* autosomal dominant inheritance, *LOF* loss-of-function, *GOF* gain-of-function, *PRCA* pure red cell aplasia

Total number of conditions for Table 10: 12

The advances in our understanding of clinical immunology continue to expand at a vast and remarkable rate, with the addition in this update of many—64, distributed across all tables (Fig. 1b)—novel genetic defects underlying inborn errors of immunity. Perhaps not surprisingly, most if not all of these new variants were identified by NGS, thus highlighting that whole exome/whole genome sequencing has become the gold standard for identifying novel pathogenic gene variants [6–8]. Indeed, since the first application of NGS to identify novel inborn errors of immunity was published in 2010 [18], ~ 45% of all currently known disease-causing variants have been discovered by whole exome/genome sequencing. Thus, a typical approach to identifying a pathogenic variant in a new patient might now consist of first sequencing a phenotype-driven panel of genes and advancing to whole exome/genome sequencing if the cause of disease remains elusive.

In this update, we increase the list of immunological diseases to 404, with 430 known genetic defects identified as causing these conditions. The unbiased application of NGS to the discovery and characterization of novel inborn errors of immunity continues to inform clinical and basic immunology. Thus, additional phenotypes have been identified for conditions resulting from variants in known and novel genes; the penetrance of genetic variants on clinical phenotypes has been shown to be highly variable; and clinical entities sharing common phenotypes have been discovered. For example, this update includes the findings that bi-allelic mutations in *ZNF341* [19, 20], *IL6ST* (encoding gp130, a common component of the receptors for IL-6, IL-11, IL-27, LIF, OSM, CNTF) [21, 22], or *IL6R* [23, 24] all cause conditions that resemble autosomal dominant hyper-IgE syndrome due to dominant negative mutations in *STAT3* [15]. Detailed analyses of these patients revealed a novel mechanism of regulating STAT3 signaling (via the transcription factor ZNF341) and defined the exact consequences of impaired IL-6/IL-6R/gp130 and putatively IL-11/IL-11R/gp130 signaling to the phenotype of AD-HIES.

Furthermore, key findings over the past 2 years continue to reveal that distinct mechanisms of disease (GOF, LOF, dominant negative, haploinsufficient), as well as different modes of inheritance (autosomal recessive, autosomal dominant) of variants in the *same* gene can cause disparate clinical conditions. This is a fascinating aspect of the genetics of human disease, and a salient reminder to be cognizant of the nature of the genetic variants identified from NGS. It is these genes that have several entries in this update. A few recent examples include:

1. Heterozygous variants in *CARD11* [25, 26] or *STAT5B* [27] can be pathogenic due to negative dominance. This was potentially unexpected because autosomal recessive LOF variants in both of these genes were previously reported to cause combined immunodeficiency and severe immune dysregulation, respectively, yet heterozygous relatives of these affected individuals were healthy [28, 29].
2. While heterozygous dominant negative mutations in *TCF3*, encoding the transcription factor E47, cause B cell deficiency and agammaglobulinemia [30], nonsense mutations in *TCF3* have now been identified that are pathogenic only in an autosomal recessive state, as heterozygous carriers of these particular allelic variants remained healthy [31, 32].
3. A heterozygous hypermorphic variant in *IKBKB* was found to cause a combined immunodeficiency [33] not too dissimilar to the original description of bi-allelic, recessive variants in *IKBKB* [34]. Similarly, bi-allelic LOF mutations in *PIK3CD* are now known to cause B cell deficiency and agammaglobulinemia [35–37], which is quite distinct from the immune dysregulated state of individuals with monoallelic activating *PIK3CD* mutations [1, 37]. This observation nicely parallels the earlier findings of either homozygous or heterozygous mutations in *PIK3R1* that clinically phenocopy recessive or activating mutations in *PIK3CD* respectively [1, 37].
4. Distinct diseases can result from heterozygous mutations in *IKZF1* (Ikaros): combined immunodeficiency due to dominant negative alleles [38] or CVID due to haploinsufficiency [39].
5. Similar to *STAT1* [40], variants in *RAC2* [41–45] or *CARD11* [25, 26, 28] can be pathogenic either as monoallelic GOF or LOF or bi-allelic recessive LOF.

Thus, these findings have revealed the fundamental importance of elucidating the impact of a novel variant on the function of the encoded protein and thus the mechanism of pathogenicity. Furthermore, these new entries are an important reminder not to overlook the potential significance of identifying heterozygous variants in genes previously believed to cause disease only in a biallelic manner or to result in a previously defined specific clinical entity. Indeed, there are now at least 35 genes that have multiple entries in the current update, reflecting the distinct mechanisms by which variants result in or cause disease (e.g., *STAT1, STAT3, NLRP1, RAC2, ZAP70, CARD11, IKBKB, WAS, JAK1, IFIH1, C3*, *C1R, C1S*–GOF or LOF; *STAT5, STAT1, CARD11, ACD, CFH, CFHR1–5, FOXN1, RAC2, TCF3, AICDA, PIK3R1, IFNGR1, TREX1, TICAM1, IRF8*–AD or AR; *PIK3CD*–AD GOF, AR LOF; *IKZF1*–AD, or haploinsufficient; *NLRP3*—distinct disease phenotypes despite all resulting from GOF alleles).

As noted above, genetic, biochemical, and functional analyses of putative novel pathogenic variants need to meet stringent criteria to be considered for inclusion in this update [17]. These criteria can make reporting genetic findings from single cases challenging, as often the best evidence that a novel variant is disease-causing is to identify additional, similarly affected but unrelated individuals with the same variants, or functionally similar variants in the same gene. While this can be challenging, particularly in light of the rarity of individual inborn errors of immunity, robust mechanistic laboratory investigations continue to provide compelling data from single patients, with or without evidence from animal models. Specifically, homozygous LOF mutations in *IRF9* [46] and *IL18BP* [47] were identified and rigorously characterized in single patients and found to be the molecular cause of life-threatening influenza and fulminant viral hepatitis, respectively.

The study and discovery of novel inborn errors of immunity can also enable improved patient management by implementing gene-specific targeted therapies. Thus, JAK inhibitors are being used to treat disorders of immune dysregulation resulting from GOF mutations in *JAK1*, *STAT1* or *STAT3* [11], while mTOR inhibitors such as rapamycin or PI3K p110δ-specific inhibitors have been reported for the treatment of individuals with *PIK3CD* GOF or *PIK3R1* LOF mutations [37]. Regarding novel gene defects, immune dysregulation due to *DEF6* deficiency was successfully treated with abatacept (CTLA4-Ig) [48]. This correlated with impaired CTLA4 expression and function in DEF6-deficient T cells [48] and parallels the therapeutic use of abatacept and belatacept for LRBA-deficiency and CTLA4 haploinsufficiency, both of which are characterized by reduced CTLA4 expression in affected regulatory T cells [49, 50]. From a theoretical perspective, the finding that MSMD can be caused by mutations in *IL12RB2*, *IL23R* or *SPPL2A* and that these mutations are associated with impaired production of IFNγ—a requisite of anti-mycobacterial immunity—implies that IFNγ administration could be therapeutically beneficial in these clinical settings [51, 52]. Similarly, recombinant IL18BP could potentially ameliorate viral-induced liver toxicity due to *IL18BP* deficiency [47].

The goals of the IUIS Expert Committee on Inborn Errors of Immunity are to increase awareness, facilitate recognition, promote optimal treatment, and support research in the field of disorders of immunity. Thus, this 2019 Update and the accompanying “Phenotypical IUIS Classification” publications are intended as resources for clinicians and researchers. Importantly, these tables underpin the design of panels used for targeted gene sequencing to facilitate genetic diagnoses or inborn errors. In the past 5 years, the number of gene defects underlying inborn errors of immunity has nearly doubled from ~ 250 to 430 (Fig. 1a). The human genome contains 1800–2000 genes that are known to be involved in immune responses [13]. Thus, the discovery and study of inborn errors of immunity has elegantly illustrated that > 20% of these immune genes play non-redundant roles in host defense and immune regulation. With the improved identification and phenotyping of patients with rare diseases, combined with high throughput genome sequencing, the number of genes fundamentally required for immunity will no doubt continue to increase, further revealing critical and novel roles for specific genes, molecules, pathways and cell types in immune responses, as well as mechanisms of disease pathogenesis and targets for immunotherapies. The field of inborn errors of immunity, and the global clinical and research communities, will therefore continue to provide key insights into basic and clinical immunology.
